# Tensor cardiography: A novel ECG analysis of deviations in collective myocardial action potential transitions based on point processes and cumulative distribution functions

**DOI:** 10.1371/journal.pdig.0000273

**Published:** 2024-08-08

**Authors:** Shingo Tsukada, Yu-ki Iwasaki, Yayoi Tetsuo Tsukada

**Affiliations:** 1 Molecular and Bio Science Research Group, NTT Basic Research Laboratories and Bio-Medical Informatics Research Center, 3–1, Morinosato Wakamiya, Atsugi-city, Kanagawa Pref., Japan Premium Research Institute for Human Metaverse Medicine (WPI-PRIMe), Osaka University, Japan; 2 Department of Cardiovascular Medicine, Nippon Medical School, Japan; 3 Department of General Medicine and Health Science, Nippon Medical School, Japan; Wake Forest University School of Medicine, UNITED STATES OF AMERICA

## Abstract

To improve clinical diagnoses, assessments of potential cardiac disease risk, and predictions of lethal arrhythmias, the analysis of electrocardiograms (ECGs) requires a more accurate method of weighting waveforms to efficiently detect abnormalities that appear as minute strains in the waveforms. In addition, the inverse problem of estimating the myocardial action potential from the ECG has been a longstanding challenge. To analyze the variance of the ECG waveforms and to estimate collective myocardial action potentials (APs) from the ECG, we designed a model equation incorporating the probability densities of Gaussian functions of time-series point processes in the cardiac cycle and dipoles of the collective APs in the myocardium. The equation, which involves taking the difference between the cumulative distribution functions (CDFs) that represent positive endocardial and negative epicardial potentials, fits both R and T waves. The mean, standard deviation, weights, and level of each cumulative distribution function (CDF) are metrics for the variance of the transition state of the collective myocardial AP. Clinical ECGs of myocardial ischemia during coronary intervention show abnormalities in the aforementioned specific elements of the tensor associated with repolarization transition variance earlier than in conventional indicators of ischemia. The tensor can be used to evaluate the beat-to-beat dynamic repolarization changes between the ventricular epi and endocardium in terms of the Mahalanobis distance (MD). This tensor-based cardiography that uses the differences between CDFs to show changes in collective myocardial APs has the potential to be a new analysis tool for ECGs.

## Author summary

Conventional analysis of electrocardiograms (ECGs) is based on its shape and the height and interval of its principal inflection points. The weighting of the waveforms and estimation of the action potential at the source of the ECG (the inverse problem) have been longstanding issues affecting the diagnosis of heart disease by ECG. Our approach is a tensor-based cardiography that uses a simple equation, the difference between two pairs of cumulative distribution functions (CDFs), to analyze the time series variance of collective myocardial APs from the ECG. By fitting this equation to the R and T waves, we obtain a set of metrics that represent beat-to-beat dynamic variance of polarization and repolarization of the epi and endocardium. Our study on ECGs from myocardial ischemia during coronary intervention shows that there are abnormalities in the tensor elements associated with repolarization, which appear earlier and more prominently than conventional changes. We believe that our approach is a revolutionary analysis tool for ECGs that holds enormous potential for clinical diagnosis, risk assessment, and prediction of lethal arrhythmias. It shows promise for cardiac disease management and has significant implications for patient care.

## Introduction

Developed by Willem Einthoven in 1901, the electrocardiogram (ECG) has been widely used in clinical practice to this day as a simple, inexpensive, non-invasive tool for evaluating the heart’s electrical phenomena [1,2]. ECG analysis is conducted in accordance with strict guidelines on voltage, width, potential and interval of the P-QRS-T wave, electrical axis, and ST segment deviation [3,4]. In clinical ECG diagnosis, classifications rely on the amplitude, interval, and morphology of the ECG’s main vertex (PQRST), as represented by the Minnesota code [5]. However, ECG diagnosis is quite difficult even for cardiologists because of the broad range of normal variance. In addition, it is difficult to clearly evaluate minute distortions on the border between a normal and abnormal ECG, which are known to be nonspecific changes related to potential heart diseases.

### A method to quantify minute changes (distortions) in ECG waveforms

In engineering, electromagnetic waves are analyzed using functional formulas like sine and delta waves [6]. A precise and efficient ECG analysis necessitates employing a suitable functional equation. Unfortunately, the intricate waveforms of ECGs have deterred widespread use of functional equations in their analysis. The lack of a model function equation that fits the ECG waveform well has limited the accuracy of thr weightings and has made diagnosis difficult. Instead of an engineering approach, measurements and analyses of ECG themselves have been used for diagnosis and risk stratification in clinical practice.

Various efforts have been made to detect abnormalities in and identify ECG waveforms. Machine learning, e.g., deep-learning methods such as continuous recurrent neural networks or long short-term memory, has also been used [7]. Deep learning of ECGs reveals that subtle distortions in ECG waveforms contain unknown features and invisible latent information related to previously unnoticed heart diseases and conditions [8]. However, these features are often difficult to explain or use as quantitative metrics.

### An equation describing the relationship between the ECG and the myocardial action potential

The relationship between the action potential of cardiomyocytes, the source of the electrocardiogram, and the electrocardiogram cannot as yet be efficiently interpreted. In particular, a method to estimate myocardial APs from ECG would be an advance in ECG-based diagnosis that could be utilized for clinical diagnosis, assessment of potential cardiac disease risk, and prediction of lethal arrhythmias. However, unlike inverse problems such as computerized tomography (CT) from X-ray radiographs and magnetic resonance imaging (MRI) from nuclear magnetic resonance, the electrocardiographic inverse problem, which is to estimate the geometric distribution of APs from the ECG, is considered difficult [9,10].

### Equation and metric of the ECG using a combination of cumulative density functions

**Fig 1:**
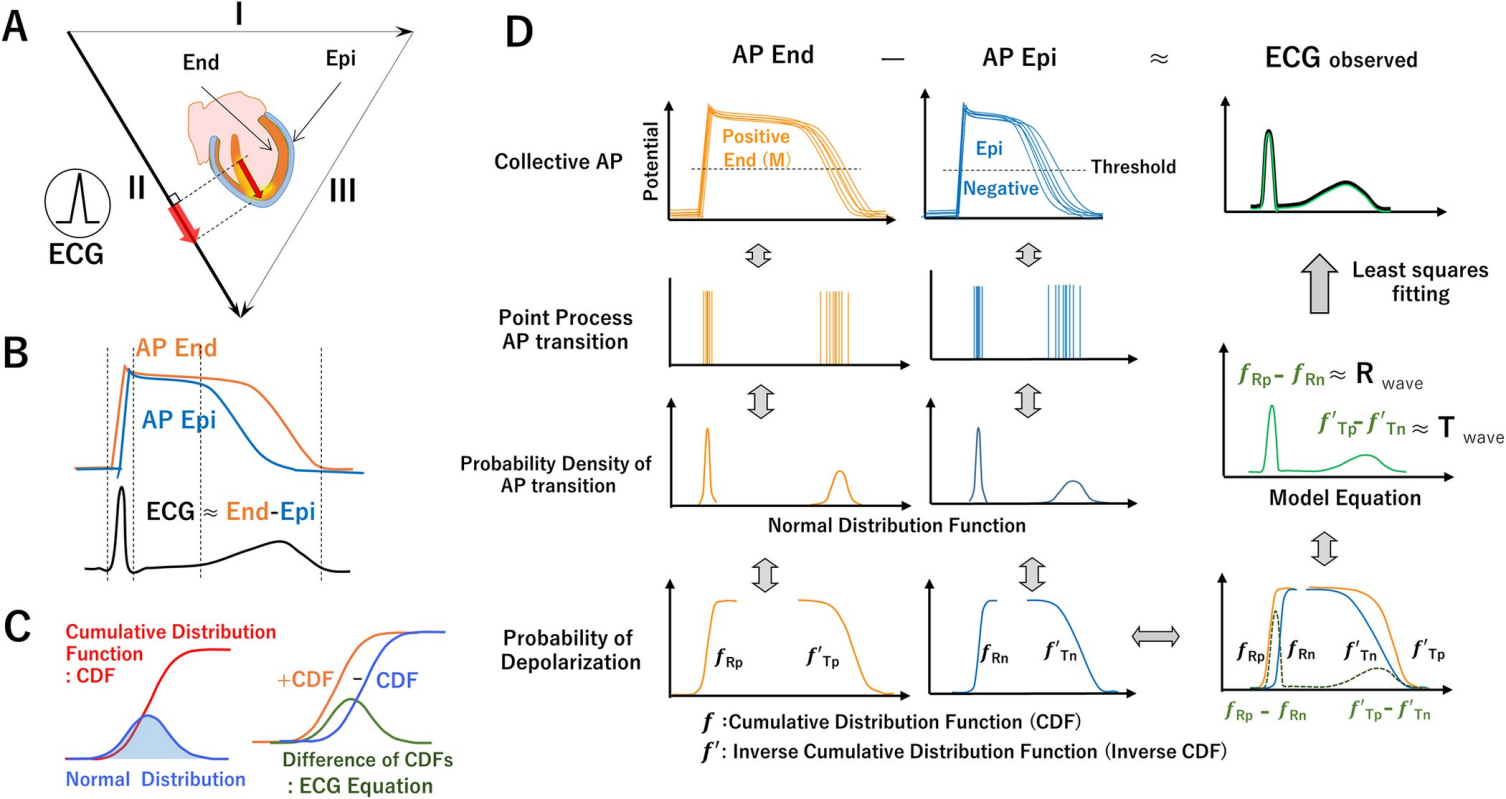
**A.** Asymmetry of ventricular wall and AP propagating from endocardial side to epicardial side and repolarization from epi to end generating R and T waves of ECG (lead II Positive Waves). **B.** Relationship between the cardiac dipole model and ECG: Depolarization starts from the endocardial side of ventricle muscle (Epi) and spreads to the epicardial side (End). Since the duration of the action potential is shorter on the Epi than the End., repolarization begins on the Epi and terminates on the End. The difference between the total potential on the Epi and that on the End of the myocardium is approximately equal to the ECG waveform. **C. Left.** Cumulative distribution function (CDF red line) and normal distribution (blue). **Right.** Equation of ECG waveform: distribution function (green line) of the difference of two CDF’s (red and blue lines). **D.** End and Epi AP elicit positive and negative potential, respectively. Their difference is closely related to ECG. ***f***: Cumulative Distribution Function (CDF) ***f***’: Inverse Cumulative Distribution Function (Inverse CDF) Timing of AP transition (threshold crossing) represented by point process, probability density (normal distributions) and probability (cumulative distribution functions, CDFs *f*_*Rp*_, *f’*_*Rn*_, *f’*_*Tp*_, *f’*_*Tn*_). Relation between end AP (f_*Rp*_, *f’*_*Tp*,_) and epi AP (*f’*_*Rn*_, *f’*_*Tp*_) and the difference in depolarization (*f*_*Rp*_- *f’*_*Rn*_) and difference in repolarization (*f*_*Tp*_- *f’*_*Tn*_) approximate the ECG R and T waves, respectively.

To address these issues, we have developed a model equation for ECG that uses the cumulative density function (CDF) difference method and point processes of collective myocardial action potentials and dipoles (Fig 1 and S1 Method). Specifically, the equation involves taking the difference between two CDFs and is used to fit the R and T waves separately (for a total of four CDFs) by using the least squares method. A fourth-order tensor is then formed by combining the standard deviation (σ), mean(μ), weights(κ), and level(β) calculated from the four CDF with the time series of heart beats(n) and the number of ECG lead channels (S1 Fig: Graphical Abstract). (Note that the tensor that is central to this method is a multi-order matrix from the informatics field, not a tensor like in electromagnetics or mechanics calculations.) The tensor indicates the variance of depolarization and repolarization of the collective myocardial anodal and cathodal APs. This method is hereafter referred to as “tensor cardiography” (TCG).

### Expected contributions and limitations of TCG analysis for cardiac disease diagnosis

TCG analysis provides a precise quantification of ECG-wave information on the APs of the myocardium in the form of probability density distributions of anodic and cathodic depolarization (R wave) and repolarization (T wave). It also has the capability of detecting cardiac abnormalities in the ECG more accurately, with greater sensitivity, and at an earlier stage than conventional methods of ECG analysis.

On the other hand, further investigations are needed in regard to whether the statistics of the collective action potentials provided by TCG are consistent with actual myocardial action potentials and whether in fact TCG is more clinically useful than conventional methods of diagnosis.

## Methods

### On the relation between ECG and myocardial APs

Previous studies have shown that the relationship between ventricular cardiomyocyte APs and ECGs is mainly formed by the asymmetric structure of the ventricular muscle and the non-uniform distribution of myocardial potentials caused by the propagation patterns of APs originating from the ventricular endocardium and reaching the epicardium, the basal to the apex [11] (Fig 1A). APs on the endocardial side of the ventricle (including middle layer, M-cell[12,13]) have a longer duration and give positive potentials to the ECG II leads, while those on the epicardial side have a shorter duration and give negative potentials to the ECG. The ECG corresponds approximately to the difference between the AP of the endocardial side and that of the epicardial side[14] (Fig 1B).

### ECG probabilistic model

The distribution of collective ventricular muscle AP transitions (the timing of the membrane potential threshold crossing) (Fig 1C) is modelled by a time-series point process (Fig 1D). The timing of the transitions is synchronized by the conduction system and has physiological variability; depolarization points are relatively densely distributed, whereas repolarization points are sparsely distributed. A normal distribution is used here, since collective myocardial AP transitions are large-scale and are often used for biomedical statistical modelling.

### Cumulative distribution function (CDF) for collective myocardial AP transitions

Ventricular muscles maintain an AP for a certain duration. Thus, the probability of a ventricular muscle transitioning from phase 0 (1) to phase 2 (ON state) can be expressed in terms of a cumulative distribution function (Fig 1D and CDF, S1 Fig). In contrast to depolarization, the repolarization state (phase 2 to phase 3 OFF state) is a process in which the probability of a ventricular muscle in the ON state progressively decreases over time. The CDF for the repolarization transition process is in the opposite direction to the time axis decreasing from 1; hereinafter, it will be referred to as the inverse cumulative distribution function (inverse CDF). It has greater variability than the CDF for the depolarization transition process.

### Difference between CDFs, ECG equation for AP transition approximation, and ECG modelling with bipolar CDFs

The relationship between the ECG and a cardiomyocyte AP is a very complex one [10,15]. To describe such complex phenomena simply, the ECG is modelled using a polarity-marked point process in which the AP exerts a positive (anodic) or negative (cathodic) binary potential change on the ECG and the sum of them corresponds to the ECG.

Specifically, the collective myocardium that contributes to the ECG of a specific lead (e.g., induction II) consists of a group of myocardium cells that produces an anodic electromotive force (EMF) on the ECG during the ON/OFF transition and a group of myocardium cells that produces a cathodic EMF.

**Fig 2:**
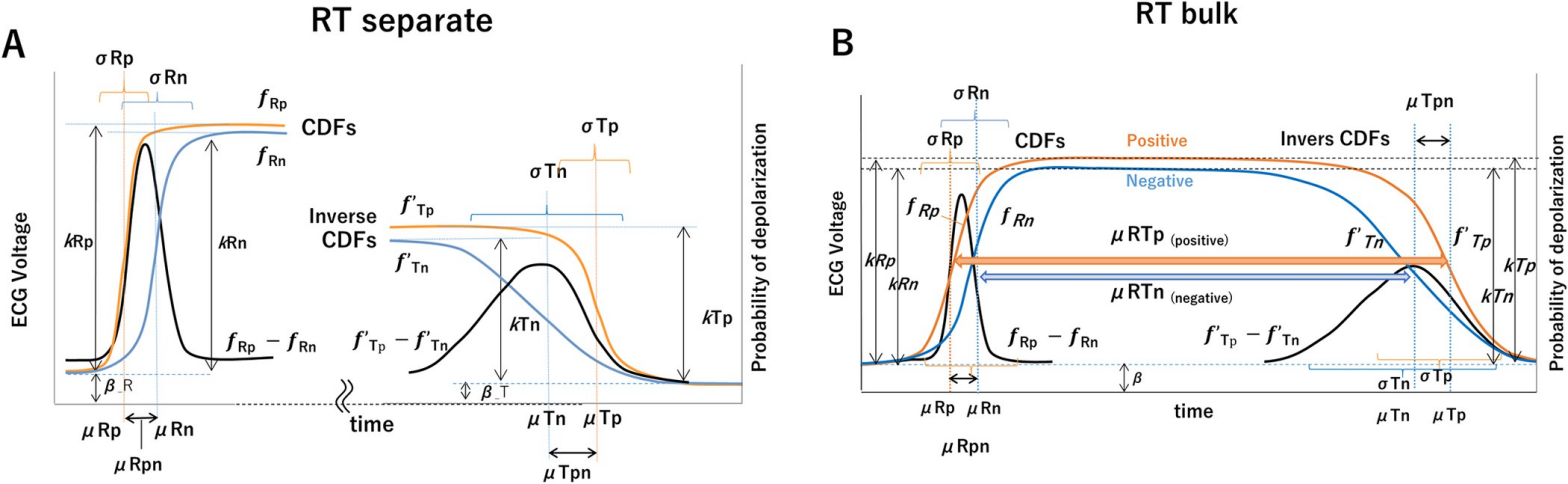
**A,** RT separation: parameters of TCG when R and T are calculated separately. **B,** RT bulk: Parameters of TCG for RT integrated calculation under the condition that APs are combined at the plateau.

Since the binary potential distributions of the anodic and cathodic groups APs are combined with opposing polarities, the difference between them can be assumed to be approximately equivalent to the ECG [16,17]. In other words, the ECG can be probabilistically represented by the difference between the transition processes of the anodic and cathodic groups by a positive—negative (anodic—cathodic) signed point process. More specifically, the collective myocardium AP transition process can be represented in terms of probability density functions by using four CDFs, two for each process of depolarization and repolarization (Fig 2A).

Here, the transition of the R wave from a resting membrane potential to depolarization is represented by an anodic and cathodic cumulative probability function (fRp and fRn, respectively).

For the ECG II lead, due to the asymmetry of the ventricle, the AP of the inner (endocardial) outer (epithelial) sides of the ventricular wall contribute to the anodal and cathodal potential, respectively (Fig 1A). According to the order of excitation, depolarization starts from the inner (anode) side of the ventricular wall and propagates to the outer (cathode) side, and the difference between the two sides of the CDF (fRp–fRn) corresponds to the ECG R wave.

The T wave is the process of transitioning from a depolarized state to a resting membrane potential. Since depolarization and repolarization are opposite in polarity, and repolarization propagates in the reverse order of depolarization due to the difference in the APD of the ventricular layer structure and function, the difference between the inner (anode) and outer (cathode) inverse CDFs (f’Tp–f’Tn) corresponds to the ECG.

The difference between the measured ECG and the equation describing the above can be minimized by using the least squares method.

The two CDFs (fR_p_ and fR_n_) and two inverse CDFs (f’T_n_ and f’T_p_) obtained by the ECG approximation respectively represent the AP-transition-process time series of the anode and cathode groups of the origin dipole of the target R and T waveforms.

Although the CDFs represent probabilities from base 0 to maximum 1, the four CDFs obtained by fitting the ECGs often result in different heights of maximum 1 (probability). Each height (i.e., weight) reveals information about the ECG R- and T-wave potentials and the combined relative anodic and cathodic potentials of the waves. The difference equations (fR_p_—fR_n_, f’T_n_—f’T_p_) are fitted independently to the QRS interval of the R wave and to the interval from the start to the end of the T wave. We will refer to this method as “RT separate” in what follows (Fig 2A).

Since depolarization and repolarization are linked with each cardiac contraction, the CDF and reverse CDF pairs for each anode (fR_p_- f’T_p_) and cathode (fR_n_ -f’T_n_) can be connected in the plateau sections by using the least squares method with the restriction that the CDF and inverse CDF are connected at the plateau (Fig 2B)_,_ To connect the anodic CDF fRp and inverse CDF fTp and connect the cathodic CDF fRn and inverse CDF fTn, constraints in the form of weights kTp and kTn are applied to equalize the T-wave height.

As a result, the probability densities of depolarization form two trapezoids with the left leg being steeper and the right leg being shallower. The level of each plateau represents a probability equal to 1 and the level of the baseline represents 0 (S1 Fig, Depolarized Probability Graph).

Converting the four CDFs into probability density functions (unimodal normal distributions) clearly shows the frequency at which the APs of the depolarizing and repolarizing anode and cathode groups transition in the time series. We will refer to this method involving conversion of the four CDFs as “RT bulk” in what follows (Fig 2B).

### ECG data from healthy participants

PhysioNet open ECG data on healthy participants in the Autonomic Aging and MIT-BIH ST change databases were used as the standard values of TCG [18,19].

### Clinical ECG data

We used two clinical ECG datasets: one on patients with effort angina and one on early repolarization syndrome. These data were obtained from the Department of Cardiovascular Medicine, Nippon Medical School.

### Statistical analysis

An ANOVA with Tukey’s honestly significant difference (HSD) test was performed. P-values lower than 0.05 were considered significant.

The Mahalanobis distance (MD) was used to represent the multivariate degree of abnormality in a time series of conventional ECG indices and new TCG indices in patients undergoing percutaneous coronary intervention (PCI). The MD is a statistical measure that has been employed in a variety of fields, including multivariate analysis of correlations in statistics, mathematical engineering, quality control and medical biology.

### Ethics statement

This study was approved by the “Central Ethics Committee of Nippon Medical School” (M-2023-159). The requirement for obtaining written informed consent was waived because it was a non-invasive, retrospective study and we used an opt-out method for participant recruitment. Additionally, verbal consent was expressly secured for the publication of the two clinical cases (case 1, case 2).

## Results

**Fig 3:**
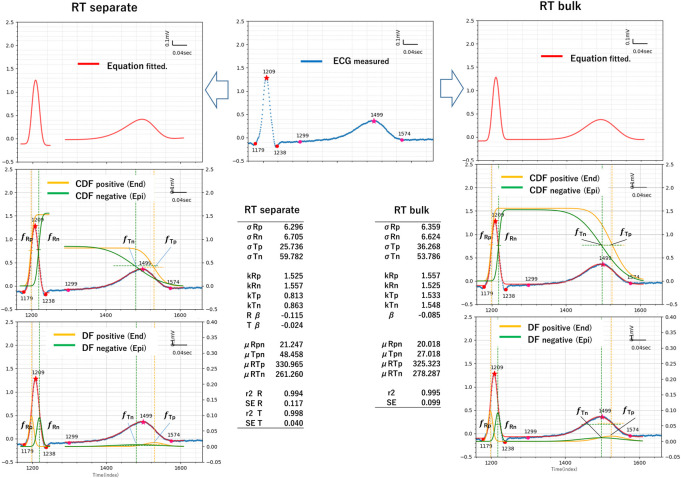
Example of TCG RT separation (left) RT bulk (right), blue dotted line: observed (target) ECG (middle), red line: TCG equation fitted to ECG minimized least square method, orange line: positive (end), green line: negative (epi). The tables (inside) show the resulting TCG parameters of RT separation and RT bulk methods.

The application of TCGA to the II-lead ECG of a healthy participant is shown in Fig 3. The P-QRS-T point was obtained from the inflection point and standard time interval (Fig 3, red points).

### RT separate method

In the RT separate method, the two CDF difference equations were fitted independently to the QRS interval of the R wave and to the interval from the start to end of the T wave. (Fig 3, blue dotted line).

Specifically, the differences, fRp—fRn and fTp–fTn, were calculated on the QRS interval of the R wave and the T-wave interval (from the start to the peak and end point of the T wave) by using the least squares method. The anodic CDF fRp and inverse CDF fTp obtained from the fitting are represented by the orange lines in Fig 3, and the cathodic CDF fRn and inverse CDF fTn obtained from the fitting are represented by the green lines. The results of the approximation equation are shown by the red line. Smooth waveforms without noise signals were obtained from the original waveforms.

The coefficients of determination (r^2^) of the QRS and T waves were 0.994 and 0.998, respectively (QRS SD = 0.117, T SD = 0.040).

The resultant four CDFs (fRp, fRn, fTp, and fTn), mean (μRp, μRn, μTp, and μTn), standard deviation (σRp, σRn, σTp, and σTn), weight (kRp,kRn,kTp, and kTn) and level (βR and βT) were obtained (Fig 3, left lower Table).

The equation for the CDF difference yields two CDFs each from the R and T waves, and in most cases, the original waveforms were well fitted, with a coefficient of determination of 0.95 or higher.

However, if the quality of the ECG signal is problematic; i.e., if the ECG contained distortion, baseline drift or noise, the fitting may fail or abnormal values might be output.

Thus, to determine the standard values of each TCG parameter and the difference between age groups, we examined PhysioNet Autonomic Aging data, which had been recorded clearly without much noise.

Table 1 shows the results of TCG performed on 699 participants selected from the ECG data of the PhysioNet Autonomic Aging database. TCG parameters were calculated for 500 heartbeats per participant and the average value was taken to be the result for each participant. The table shows the mean values and standard deviations for the 699 participants.

**Table 1:** TCG parameters by age group using RT separate method.

Age	kRp	kRn	kTp	kTn	σRp	σRn	σTp	σTn	μRTp	μRTn	μRpn	μTpn
**All Ave**	1.99	2.01	0.8	0.8	6.71	5.83	23.56	54.29	307.35	231.7	22.18	53.48
**SD**	±0.66	±0.62	±0.32	±0.32	±1.05	±1.5	±2.68	±9.48	±23.67	±26.89	±3.26	±10
**18–19**	1.85	1.86	0.71	0.71	6.28	5.45	22.75	54.6	305.4	228.78	20.59	56.04
**20–29**	2.07	2.09	0.83	0.83	6.71	5.74	23.49	54.37	306.76	231.2	22.3	53.25
**30–39**	1.89	1.88	0.76	0.77	6.64	6.1	23.71	53.09	305.33	229.38	21.87	54.07
**40–49**	1.82	1.81	0.65	0.68	7.01	6.35	23.63	54.59	307.43	230.11	22.64	54.69
**50–59**	1.73	1.73	0.72	0.77	7.29	6.17	24.8	55.38	321.33	246.68	23.11	51.54
**60–69**	1.43	1.47	0.57	0.62	6.45	5.72	25.16	56.55	328.36*	259.69*	21.31	47.36

(*P<0.05, compared to 20–29, 30~39)

The time series is in the order of μRp, μRn, μTn, and μTp. The standard deviations σRp, σRn, σTp, and σTn are distributions (time series widths), and they follow the relationship σRn<σRp<< σTp<σTn. The heights kRp, kRn, kTp, and kTn represent the relative weights of each function.

Most of the TCG parameters were stable as to their correlation and showed little change with age. However, the time-related TCG parameters showed significant differences; i.e., a prolongation of μRTp and μRTn and a decrease in μRTp and μRTn were observed in the 60-69y group (Table 1).

### RT bulk method

The RT separate method yielded different heights for the four CDFs of the TCG, and the CDF and inverse CDF were not connected. Therefore, to connect the anodic CDF fRp and inverse CDF fTp, and the cathodic CDF fRn and inverse CDF fTn, respectively, constraints were applied to equalize the T-wave height by using the weights kTp and kTn (RT bulk method, Figs 2B and 3, right side).

The coefficient of determination (r^2^) of the bulk method was 0.995 (bulk method SD = 0.099).

The RT separate and bulk methods gave slightly different means, standard deviations, and weights (Fig 3 right side, Table 2). The results of the bulk method tended to fit broadly in the time axis direction, including the ST level, while those of the RT separate method were in the vertical amplitude direction of the R and T waves. This difference was believed to have been caused by the different fitting areas of the R, T and ST waves.

**Table 2:** TCG parameters by age group using RT bulk method.

Age	kRp	kRn	kTp	kTn	σRp	σRn	σTp	σTn	μRTp	μRTn	μRpn	μTpn
**All Ave**	1.97	1.88	1.94	1.91	6.56	5.51	35.73	50.07	299.98	256.13	20.99	22.86
**SD**	±0.61	±0.58	±0.6	±0.59	±1.01	±1.66	±5.24	±7.3	±24.62	±23.72	±3.28	±9.55
**18–19**	1.83	1.75	1.8	1.78	6.18	5.14	35.75	50.11	298.13	255.44	19.48	23.21
**20–29**	2.04	1.94	2.01	1.97	6.56	5.39	35.5	50.26	299.65	255.53	21.05	23.07
**30–39**	1.86	1.78	1.83	1.81	6.43	5.82	35.46	49.41	297.89	253.98	20.93	22.98
**40–49**	1.81	1.75	1.78	1.77	6.87	6.15	37.23	49.81	298.86	256.89	21.64	20.33
**50–59**	1.73	1.67	1.7	1.7	7.12	6.01	37.07	49.83	312.25	266.65	21.89	23.71
**60–69**	1.47	1.45	1.46	1.46	6.61	5.84	39.1	48.84	318.07*	278.89*	20.05	19.13

(*P<0.05, compared to 18~19, 20–29, 30~39,40~49)

The correlation coefficients between the TCG parameters with relation to time (interval), QT, and R-R interval (RRI) are shown in S1A Table, and the correlation coefficients for the TCG parameters related to potentials are shown in S1B Table (the scatter plots are shown in S2 Fig).

QT was correlated with μRTp (r = 0.88) and μTpn (r = 0.74), RRI was correlated with μRTp (r = 0.73), and μRTp was correlated with μRTn (r = 0.66). kRp and kRn were highly correlated with each other (r = 0.98) and with the height of the R wave. kTp and kTn were highly correlated with each other (r = 0.95) and with the height of the T wave (r = 0.74, r = 0.67 respectively). The ST level was correlated with Tβ(r = 0.80), and Rβwas correlated with Tβ(r = 0.65).

### Clinical application of TCG: case report presentation

#### Case 1: A male patient with angina underwent percutaneous coronary intervention (PCI) for left anterior descending artery (LAD)

We investigated the relationship between the ECG changes and TCG parameters during elective PCI for 90% stenosis at the left anterior descending coronary artery (#6–7). The patient showed ST level changes with transient myocardial ischemia induced by three balloon inflations.

**Fig 4:**
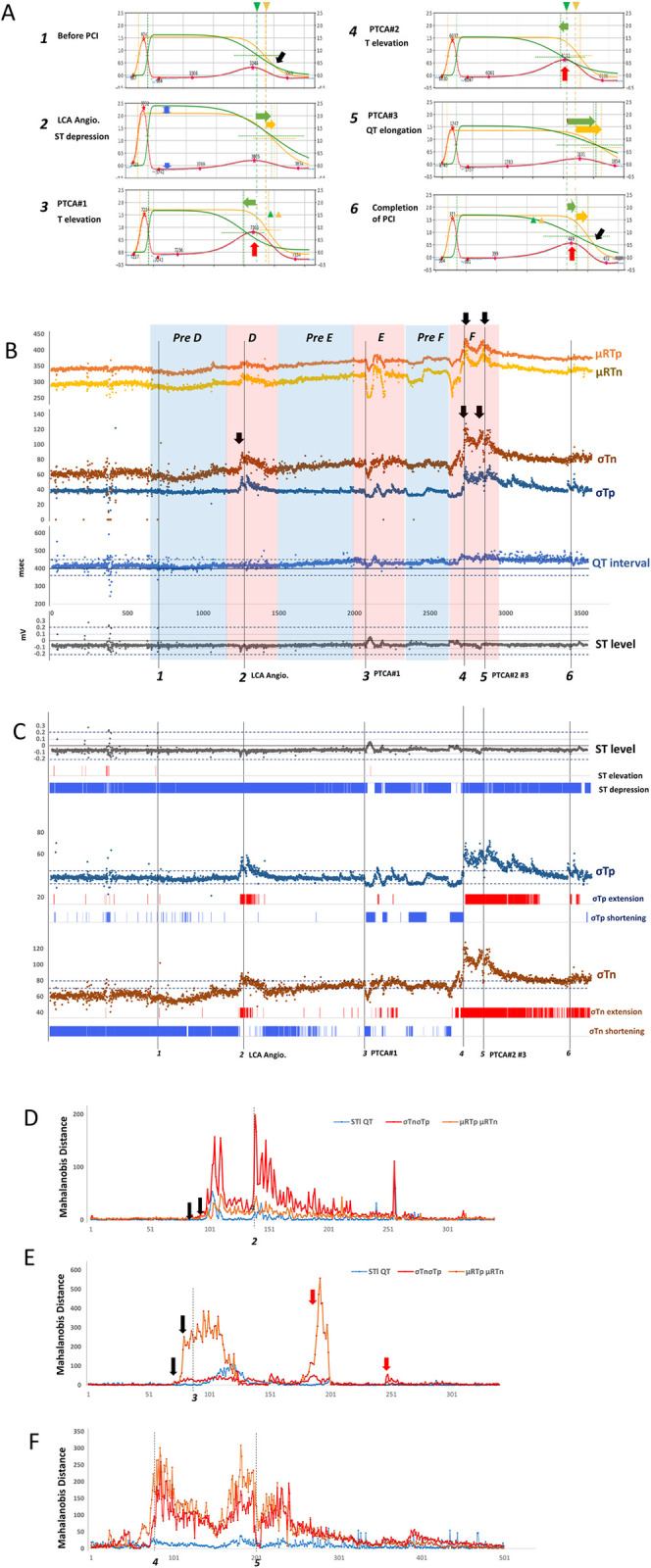
TCG parameters of patient with angina during elective PCI. **A,** Graphs of TCG f(RT bulk) equation fitted to ECG measured during the intervention ***1*.** Before PCI, ***2***. LCA angiography: ST depression, ***3***. First PTCA: T elevation, ***4***. Second PTCA: T elevation ***5***. Third PTCA: QT elongation, ***6***. Completion of PCI: T elevation, (***1~6*** locations indexed in **B**). Blue dotted line: target ECG, Red line: fitting of TCG equation to ECG, Orange line: positive (end) CDF, Green line: negative (epi) CDF; the crossings of the vertical and horizontal straight dashed lines represent the locations of the mean of CDF. Arrow heads mark the original locations (***1***) of the mean. Horizontal arrows (green and yellow) indicate repolarization time shifts during PCI. The red arrow indicates T-wave elevation. Black arrows indicate crossings of the epi end inversion of repolarization. **B**, Time-series graph illustrating TCG time-related parameters (μRTp, μRTn, σTp, and σTn) alongside conventional ST level and QT interval during the intervention. The numbers at the bottom indicate times corresponding to TCG graphs in **A**. Black arrows highlight points not detected by conventional indicators but identified by TCG parameters. **C**, Threshold for evaluation of ischemia by conventional criteria is an ST elevation of +0.5 mV and ST depression of -0.5 mV. In this case, there was a persistent ST depression of about -0.5 mV before and during PCI (Upper, blue, ST depression). The visible ST changes due to PCI (PTCA #1 and #2) were taken to indicate the disappearance of ST depression. No ST change was observed in CAG or PTCA #3. ST elevation (+0.5 mV) due to PCI was not determined. Time-series changes in the parameters of TCG, σTp (middle) and σTn (bottom). Extension or shortening of σ caused by the PCI were determined by fixed thresholds (σTp ≥ 45 elongation: marked red, σTp ≤ 35 shortening: marked blue). Extension and shortening of σTn thresholds were ≥ 80 (marked red, bottom) and ≤ 70 (marked blue), respectively. **D**, Mahalanobis distance (MD, a multivariate statistical abnormality) of conventional indexes ST level and QT interval (blue line) and TCG parameters σTn and σTp (red line), μRTp and μRTn (orange line) between the intervals of **pre D** (reference) and **D** (target), marked as blue and red rectangles in **B**. **E**, MD between **pre E–E**. **F**, MD between **pre F–F**.

As shown in Fig 4A, in the resting ECG before balloon inflation, the ST level was normal (ECG V5 lead, blue dotted line). The results for the bulk method indicated that the heights of the cathodic and anodic plateau levels were almost equal. The cathodic ***f***Tp(green lines) represented elongation of the repolarization, resulting in the ***f***Tp and ***f***Tn crossing (black arrow).

During the left coronary angiography with contrast medium, the ECG showed ST depression and an increase in R waves indicating myocardial ischemia. The TCG showed that ***f***Rn was markedly elevated compared with ***f***Rp. In addition, a backward shift of ***f***Tp and ***f***Tn was observed (Fig 4A 2, orange arrow).

Transient myocardial ischemia was induced by a coronary artery occlusion due to the balloon inflation and resulted in an increase in T-wave amplitude on the ECG (Fig 4A 3, red arrow); TCG showed an enlargement of the gap between ***f***Tp and ***f***Tn due to a forward shift of ***f***Tn (green arrow).

The second balloon inflation also elicited an increase in the T waves on the ECG (Fig 4A 4). The TCG results indicated marked elongation of cathodal repolarization (green line, downward slope).

The third balloon inflation resulted in a prolonged QT on the ECG; TCG showed a marked backward shift in ***f***Tn and ***f***Tp (Fig 4A 5, orange and green arrows).

After completion of the PCI, the ST depression disappeared from the ECG. TCG results showed ***f***Rp and ***f***Rn (***f***Tp and ***f***Tn) with equal heights (Fig 4A 6). The time direction shift of ***f***T was also reduced, but the ***f***Tp and n***f***Tn crossings remained (black arrow).

Fig 4B shows a time-series graph of the TCG time-related parameters, μRTp, μRTn, σTp, and σTn, and the conventional ST level and QT interval during the intervention. σTn,σTp, μRTn, and μRTp showed markedly fluctuated and long-lasting responses (black arrows).

Table 3 lists the TCG parameter values at time points *1–6*. Bolded values indicate notable values at each time point from A to F.

**Table 3:** TCG parameters during PCI for LAD (Time points 1–6 in Fig 4).

	kTp	kTn	σTp	σTn	μRpn	μTpn	μRTp	μRTn
***1***	1.58	1.59	37.48	60.39	23.77	23.62	336.25	288.85
***2***	**2.16**	**2.38**	**56.72**	86.59	26.70	**7.77**	360.74	326.27
***3***	**1.78**	1.58	32.35	57.59	23.25	**67.43**	341.00	**250.32**
***4***	1.59	1.47	31.63	66.97	23.91	**52.34**	350.72	274.47
***5***	1.36	1.54	**54.18**	**104.01**	25.59	14.32	**408.53**	**368.62**
***6***	1.69	1.69	31.81	87.27	23.05	30.60	368.83	315.18

ST depression (2) affected the bulk σTp, σTn, and μTpn. T-wave amplitude increased μTpn (*3*, *4*) and kTp (*3*), QT elongation (*5*) elongated σTn, σTp, μRTp, and μRTn (Table 3).

As described above, specific parameters of TCG varied with ST-T changes associated with ischemia in PCI. The characteristics of the ECG were explicitly quantified for each parameter.

The alterations induced by PCI in the TCG parameters (σTn and σTp; μRTp and μRTn) and ECG parameters (ST level and QT interval) were assessed by making a simple threshold judgement using conventional criteria[20] and the Mahalanobis distance, a statistical multi variance abnormality index.

According to the 4^th^ universal definition of myocardial infarction, the threshold for evaluation of ischemia by conventional criteria is an ST elevation of +1.0 mV and ST depression of -0.5 mV[21]. In this case, there was a persistent ST depression of about -0.5 mV before and during PCI (Fig 4C Upper, blue, ST depression). Therefore, the visible ST changes due to PCI (PTCA #1 and #2) were determined to be the disappearance of ST depression. No ST change was observed in CAG or PTCA #3. ST elevation (+0.5 mV) due to PCI was not determined by conventional criteria in this case (Fig 4C Upper, Red).

The TCG parameters, σTp (Fig 4C middle) and σTn (Fig 4C bottom) represent the standard deviation of the anodic and cathodic aspects of repolarization, respectively. The σ standard deviation could be lengthened or shortened depending on the PCI. CAG and PTCA #1 and #3 were determined as the elongation of σTp, while PTCA #2 was judged as shortening. Similarly, σTn showed similar lengthenings and shortenings of σTp. σTn was more unstable than σTp and showed a persistent change induced by repetitive PTCA.

Due to PCI’s impact on basal coronary blood flow and the ECG, MD was calculated using the interval immediately before PCI as a reference. As shown in σTn in Fig 4C, the ECG still showed changes due to PCI, thus we divided the interventions into segments (D-F) and calculated the abnormality from the waiting periods prior to each intervention (PreD-F).

To compare the TCG results (σTn and σTp—red line, μRTp and μRTn—orange line in Fig 4D, 4E and 4F) with conventional indices of ischemia (ST level and QT interval—blue line Fig 4D, 4E and 4F), the Mahalanobis distances (MD, a multivariate statistical abnormality, S3 Method) were calculated during the intervention (Fig 4B, red rectangles D, E, F). These distances were determined from the distribution during the pre-intervention wait (Fig 4B, blue rectangles, pre D, pre E, pre F) and time-series graphs of MD were obtained (Fig 4D, 4E and 4F).

In the presence of LCA angiography (2), the MD of the TCG index increased more than 10 heartbeats earlier than in the conventional index, and with greater distance and higher elevation (Fig 4D). During PCI-induced ischemia, the MD of the TCG index rose more than 20 heartbeats earlier than the conventional index (Fig 4E, black arrows). In the late phase of PCI, the TCG index exhibited a greater distance than the conventional index (Fig 4F). The small transient elevations of the TCG index (red arrows), not observed in the conventional index, suggested that TCG might be able to detect minor ischemic responses not represented in conventional indexes (Fig 4E and 4F).

#### Case 2: Early repolarization syndrome (ERS) presenting ventricular fibrillation

An early repolarization electrocardiographic pattern is characterized by a sharp, well-defined positive deflection or notch immediately following a positive QRS complex at the onset of the ST segment or slurring at the terminal portion of the QRS complex. An imbalance in ionic current between the epicardium and endocardium in the ventricle can lead to the development of a transmural voltage gradient that might manifest as an early repolarization (ER) ECG pattern. An ER-ECG pattern has been confirmed in 6–24% of the general population[22,23,24]. The prognoses of the majority of the participants found in the database with an ER-ECG pattern were considered to be benign. However, a relationship between ER-ECG and sudden cardiac death in a number of the patients showing an ER-ECG pattern has been reported. Risk stratification of the ER-ECG pattern is an important issue in clinical practice to prevent sudden cardiac death. Because of the high prevalence of the ER-ECG pattern in the general population, previous studies have shown that it is difficult to distinguish between malignant and benign types of ER-ECG patterns by using 12-lead ECG.

In this case study, a patient (age in their 20’s) with early repolarization syndrome (ERS) who had a history of multiple repetitive episodes of ventricular tachycardia or ventricular fibrillation (i.e., electrical storm) was tested using TCG. Early repolarization pattern was confirmed in inferior leads and lateral leads. However, there were no significant ECG parameter abnormalities including ST-T changes, QRS axis deviation, or QT interval.

A subcutaneous implantable cardioverter-defibrillator (ICD) was implanted because of the patient’s history of ventricular fibrillation with no structural heart disease. The ECG showed an ER-ECG pattern in inferior-lateral leads and diagnoses as ERS.

**Fig 5:**
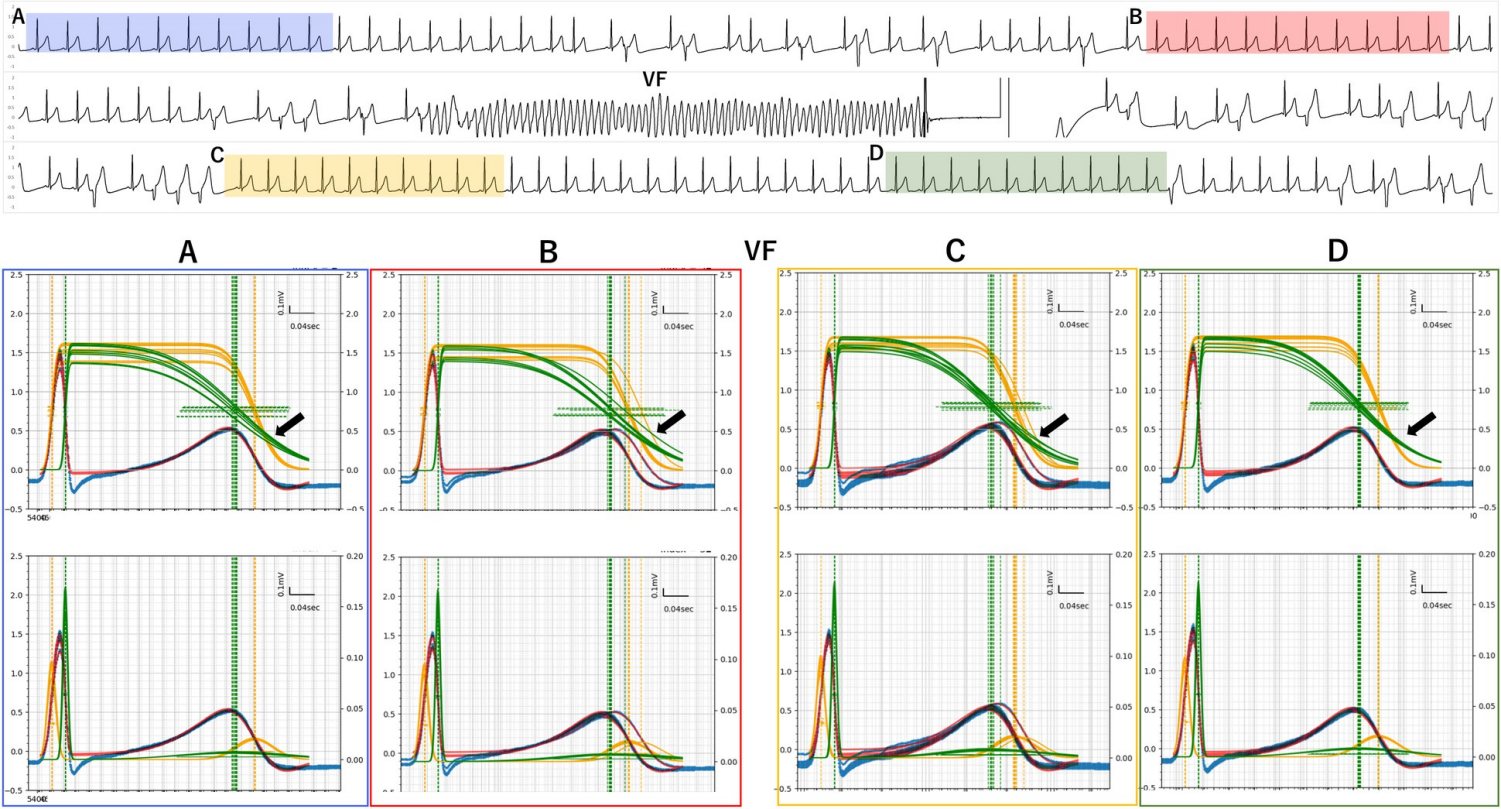
TCG results of malignant early repolarization syndrome (ERS) presenting ventricular fibrillation (VF). Top ECG (V6) of 50 sinus rhythm pulses prior (**A** blue), 20 pulses before (**B** red), 20 pulses after (**C** yellow), and 45 pulses after (**D** green) VF. Bottom 10-pulse overlays of TCG of A~D sections. Black arrows indicate where fTn and fTp reversed and had marked elongations and variance of μTn. Red arrows indicate alternans of T waves.

The ECG (V6) of 50 sinus-rhythm pulses prior to ventricular fibrillation (VF) was apparently normal (Fig 5A). The TCG (RT Bulk method) showed that the relative positions of ***f***Tn and ***f***Tp were reversed and crossed (Fig 5A, black arrow) at the end of the T wave due to marked elongation of μTn (Fig 5A, black arrow indicating green lines).

The CDF graph of a 10-pulse overlay (Fig 5B) and animation (S3 Fig) showed marked variance in the repolarization phase. The twenty pulses before (B) and after (C) ventricular fibrillation showed alternans of T waves with marked variance in ***f***Tn (Fig 5B and 5C, black arrows indicating green lines) compared with the 45 pulses after VF (D).

A ***f***Tn—***f***Tp crossing and ***f***Tn—***f***Tp reversal were still present before VF and after returning to sinus rhythm (Fig 5C and 5D).

Of the TCG parameters, μTn, Tpn, and cRTp were significantly increased in ERS (Table 4). This finding is adapted to the characteristic of ERS. Not only was the onset of repolarization accelerated, but also a delay of repolarization termination was suggested. Two or three patterns of repolarization represented the repolarization complexity of the ER-ECG. These results show that TCG can be used to evaluate epicardial and endocardial features in the patients with an ER-ECG pattern to potentially predict high risk patients.

**Table 4:** TCG parameters for every 10 sequential beats of the ERS patient (Fig 5A, Section A-D).

Pulses	σRp	σRn	σTp	σTn	μRTp	μRTn	μRpn	μTpn	kRp	kRn	kTp	kTn	β	RRI	QT
**A1**	6.9	3.76	28.29	89.01	325.8	266.65	22.15	36.99	1.38	1.36	1.37	1.36	-0.08	820	384
**A2**	6.7	3.8	29.87	86.61	324.23	271.72	21.83	30.68	1.54	1.53	1.53	1.53	-0.06	822	381
**A3**	6.67	3.83	30.24	86.67	324.51	273.64	21.71	29.16	1.6	1.59	1.59	1.59	-0.05	819	379
**A4**	6.7	3.75	30.31	85.65	324.46	272.25	21.72	30.49	1.61	1.59	1.59	1.6	-0.06	822	379
**A5**	6.73	3.82	30.68	85.22	324.54	274.92	21.65	27.97	1.63	1.62	1.62	1.62	-0.06	820	377
**A6**	6.72	3.71	29.69	87.1	325.22	270.72	21.86	32.64	1.5	1.48	1.49	1.48	-0.06	820	382
**A7**	6.95	3.82	29.01	88.76	325.57	266.83	22.12	36.62	1.4	1.37	1.39	1.37	-0.08	816	384
**A8**	6.64	3.76	29.8	86.35	324.06	268.54	21.9	33.62	1.52	1.5	1.51	1.51	-0.06	818	386
**A9**	6.79	3.83	30.45	85.48	324.22	272.67	21.79	29.76	1.6	1.59	1.59	1.59	-0.06	819	385
**A10**	6.74	3.8	30.64	85.96	324.26	272.48	21.72	30.06	1.61	1.6	1.6	1.61	-0.06	819	383
**B1**	6.86	3.91	32.58	87.23	345.58	297.54	21.84	26.21	1.55	1.54	1.54	1.54	0.01	804	424
**B2**	6.67	3.76	31.11	89.16	327.44	276.39	21.78	29.27	1.41	1.39	1.4	1.4	-0.06	806	391
**B3**	6.71	3.78	30.14	87.97	326.81	273.13	21.88	31.8	1.44	1.42	1.43	1.42	-0.07	809	386
**B4**	6.72	3.84	31.29	85.7	325.52	276.36	21.64	27.51	1.58	1.56	1.57	1.57	-0.05	811	384
**B5**	6.71	3.84	31.47	85.49	325.44	276.35	21.57	27.52	1.61	1.59	1.6	1.6	-0.06	815	390
**B6**	6.71	3.77	31.08	85.57	325.28	276.18	21.62	27.48	1.61	1.59	1.59	1.59	-0.05	819	384
**B7**	6.71	3.74	30.84	83.99	325.21	274.56	21.59	29.07	1.59	1.56	1.58	1.57	-0.07	824	383
**B8**	6.75	3.72	29.9	88.56	326.2	272.98	21.83	31.39	1.46	1.44	1.45	1.44	-0.05	829	381
**B9**	6.91	3.78	29.08	87.6	326	270.41	21.89	33.7	1.44	1.42	1.43	1.42	-0.08	829	382
**B10**	6.79	3.79	30.34	85.11	325.15	274.4	21.7	29.04	1.58	1.56	1.57	1.57	-0.06	834	383
**C**1	7.63	4.1	31.25	82.52	324.41	265.72	21.92	36.77	1.57	1.53	1.56	1.53	-0.07	741	453
**C2**	7.08	3.9	29.51	78.84	310.85	249.05	21.9	39.91	1.58	1.54	1.58	1.54	-0.14	736	390
**C3**	7.06	3.9	30.49	78.34	309.73	251.28	21.73	36.72	1.67	1.64	1.67	1.64	-0.16	734	387
**C4**	6.34	3.58	31.06	92.84	312.94	254.3	21.78	36.86	1.59	1.56	1.58	1.57	-0.1	733	441
**C5**	6.87	3.88	30.72	78.57	309.09	250.75	21.63	36.71	1.69	1.67	1.68	1.67	-0.15	731	377
**C6**	6.69	3.91	30.71	74.94	307.97	246.32	21.58	40.08	1.69	1.68	1.68	1.68	-0.13	731	374
**C7**	6.96	3.85	30.34	78.6	309.6	250.31	21.82	37.47	1.6	1.58	1.59	1.58	-0.11	734	383
**C8**	7.19	3.89	30.19	79.96	310.64	251.11	21.97	37.55	1.53	1.49	1.52	1.49	-0.14	735	434
**C9**	7.09	3.96	30.63	76.96	309.11	250.91	21.86	36.34	1.61	1.58	1.61	1.57	-0.17	736	387
**C10**	6.98	3.92	31.26	78	309.12	253.3	21.73	34.1	1.68	1.65	1.67	1.65	-0.15	738	376
**D**1	6.87	3.92	31.72	77.88	309.79	256.19	21.76	31.84	1.66	1.65	1.66	1.65	-0.11	750	372
**D2**	6.83	3.92	31.61	77.84	309.98	256.91	21.67	31.4	1.68	1.67	1.67	1.67	-0.09	749	383
**D3**	6.92	3.89	31.77	76.3	309.57	256.69	21.59	31.29	1.7	1.68	1.69	1.68	-0.11	751	372
**D4**	6.92	3.86	31.27	78.71	310.48	256.68	21.73	32.06	1.61	1.59	1.6	1.59	-0.1	753	372
**D5**	7.08	3.89	30.86	80.64	311.25	255.86	21.94	33.45	1.51	1.5	1.51	1.49	-0.1	757	386
**D6**	6.95	3.9	30.9	79.59	311.03	255.56	21.86	33.61	1.56	1.55	1.55	1.55	-0.12	757	374
**D7**	6.93	3.93	31.74	76.74	310.06	258.64	21.71	29.72	1.66	1.65	1.66	1.65	-0.11	758	371
**D8**	6.86	3.94	31.84	77.62	310.25	258.02	21.58	30.65	1.67	1.66	1.67	1.66	-0.08	759	373
**D9**	6.84	3.9	31.83	78.38	310.69	259.24	21.6	29.85	1.69	1.68	1.68	1.68	-0.07	760	370
**D10**	6.96	3.91	32.06	77.66	310.64	259.88	21.54	29.21	1.7	1.67	1.69	1.67	-0.09	763	369

The cause of the ER-ECG pattern is associated with the transmural voltage gradient between epicardium and endocardium at the early repolarization phase. An experimental study suggested that heterogeneous loss of the AP dome produces phase-2 re-entry, leading to VF [25]. However, the exact mechanism of VF development in patients with ERS is still uncertain. T-wave alternans reflects sudden changes in temporal heterogeneity in ventricular repolarization and is a sign of cardiac instability leading to life-threatening arrhythmia [26]. In addition, microvolt T-wave alternans have been confirmed in patients with ERS [27]. In the present case, TCG showed the dynamic nature of repolarization alteration mimicking T-wave alternans between epi and endocardial lesions prior to VF development. However, such unique behavior of transmural alteration during the repolarization phase was not revealed by the 12-lead ECG. This case study hence shows that TCG can potentially represent the beat-to-beat transmural vulnerability at the repolarization phase leading to VF.

## Discussion

### Mathematical ECG modelling and analysis

The van der Pol oscillator and Nagumo models serve as mathematical formulations of the electrocardiogram (ECG), and they have been used in the development of ECG wave generators [28,29,30]. The application of Fourier and wavelet transforms to ECG frequency analysis reveals the spectral characteristics of ECG signals. Notably, recent advances have involved using machine learning for generation and classification of ECG [8,31]. However, mathematical models specifically designed to analyze myocardial population action potentials, their duration (APD), and cardiac electromagnetic field (EMF) dipoles have not been developed

### The electrocardiogram inverse problem

The quest to decipher the electrical activity of the myocardium, the origin of the ECG signal, stands as a pivotal inverse problem in the realms of physiology and medicine. Over the course of history, numerous methodologies have been explored to address this intricate challenge. Termed an ill-posed problem, its stable resolution is hindered by the substantial asymmetry of information, the spatio-temporal intricacies of myocardial electrical activity, the nonlinear and non-uniform propagation of current in organs and tissues, and the unique anatomical and physiological attributes of the heart and thorax [9]. Consequently, solutions to this problem have been pursued through the employment of simplistic models like dipoles and cardiac surface potential distributions. Alternatively, researchers have introduced various constraints grounded in the anatomy and physiology of the heart [32,33].

Recently, finite element simulations of the heart have been used to model the potential of myocardial tissue [15,34]. This involves making a bidomain model representing the membrane and extracellular potential of myocardial cells at numerous points [35].

Constrained bipolar and cardiac surface potential models, along with finite element cardiac simulations, play a role in defibrillator development and analyzing abnormal waves related to fibrillation [36,37]. However, their application to general clinical ECG diagnosis remains limited.

Recent engineering efforts to address the inverse ECG problem have prioritized the spatial distribution of myocardial action potentials over their temporal distribution. Nevertheless, recognizing the temporal distribution of action potentials in medical ECG analysis could be advantageous for individual patient diagnosis. As a result, our focus in this study was on determining the collective state of myocardial action potentials from ECG, with a priority on the temporal distribution.

### Method of estimating the distribution of point processes of collective myocardial AP transitions from the ECG

An ECG is generated by a complex system of biological and biophysical mechanisms. We alleviated the problem with the relationship between the ECG and the electromotive force of the myocardium by modeling the timing of the AP transitions and polarity (anode/cathode to ECG) in collective myocardial AP transitions as a point process. Differences in myocardial APs, e.g., differences in AP propagation in myocardial fibers in cardiac electrophysiology experiments [14,38], differences in cross-layer potentials in wedge-shaped myocardium [39], and differences between monophasic APs and location-independent signals in cardiac electrophysiology simulations [17], are closely related to the ECG. We prepared two CDFs to represent the transition process from the resting membrane potential to depolarization of the ventricular muscle: one representing the transition process of the ventricular muscle collective that is positive to the ECG and the other negative. On the basis of their inverse relationship as dipoles, the difference between the two forms an ECG-equivalent equation, and the parameters of these functions approximate those of the ECG. In most cases, the coefficient of the equation determination to the ECG waveform is greater than 0.95.

An approximate equation using the difference between the two CDFs to fit the R and T waves calculates four CDFs with metrics of mean, standard deviation, weights, and levels. Combining the metrics with the time series of beats and the number of channels in the ECG leads forms a fourth-order tensor. The elements of the tensor and the time-series graph of the four CDFs indicate the variance of collective myocardial AP transitions.

The ECG source, cardiac EMF, is thought to be caused by either the asymmetric shape of the heart (hemispheric shape of ventricular wall, without the myocardial wall on the basal portion) or complex patterns of polarization of the myocardium (depolarization starts from the endocardial side and propagates to the outer side, and after maintaining the depolarized state, repolarization starts from the outer side and ends on the inner side). If these patterns are normal, the anode positive myocardium group tends to be distributed on the endocardial side and the cathode negative group distributed on the epicardial side. In cardiac physio pharmacology studies, M cells have been proposed as a group of cardiomyocytes with long APDs; the TCG parameters of **Tp** (**kTp, σTp, μRTp, μTpn**) are thought to correspond to repolarization of M cells [12,39].

### AP duration and TCG parameters, μRTp, μRTn

The value of μRTp, the average of the anodic CDF from start to end, represents the collective AP duration and it is close to the reported APD value [40,41]. In contrast, the μRTn cathodic collective APs is shorter than that of APD (Table 1 RT separate,μRTn, 18–19). The presence of an early repolarizing group in the cardiomyocyte collective and a gradual decrease in the plateau potential of the AP to repolarization may shorten the length of μRTn more than that of APD. In fact, shortening of μRTn is frequently observed in myocardial ischemia. This is consistent with the shortening or loss of the plateau of the myocardial AP and the rapid descent of the potential after depolarization (triangular wave) in ischemic early repolarization[42,43]. In the clinical ECG cases presented above, abnormal TCG patterns appeared at both the positive and negative poles. In such cases, the repolarization-negative component, Tn (μRTn,σTn, kTn) tends to be more sensitive to abnormalities of the Phase 2 repolarization than the Epi: negative to End: positive relation of repolarization. Thus, our findings suggesting that Tn(μRTn,σTn, kTn) may be useful as a common marker of abnormalities of cardiac repolarization. It should be noted that abnormal ECGs with conduction disturbances or extra systoles might disrupt the relationship between the action potentials and distribution of point process.

As an aging-related change in TCG parameters, prolongations of μRTp and μRTn were observed in the elderly group, suggesting an association with the previously reported elongation of APD in aged cardiomyocytes [44,45,46].

### Characteristics of the RT bulk and separate methods

The RT bulk method was introduced to improve visibility by connecting the y-axis of the CDFs and inverse CDFs of the R and T waves in two phases with their heights aligned, creating a trapezoidal shape similar to an AP. As a result, by connecting the anodal and cathodal components of the R and T waves with their respective aligned heights, it becomes possible to express the relationship of the plateau of AP positive and negative myocardial groups separately. The RT bulk method is particularly suitable for analysis of ischemic heart disease, where ST changes are important. In particular, the T-wave-height equalization constraint increases the stability of the TCG calculation, preventing abnormal results due to fitting errors when ECG contains noise or distortion. However, it was also found that, by equalizing the he T-wave heights (T_k_), the RT bulk method may cause distortion and suppression effects on the time axis, such as to the mean and standard deviation of the CDF, and may give different results from the RT separate method. In such cases, the results of the RT separate method would be correct. Therefore, it is considered desirable to choose the best method for the application. In the case studies, the RT bulk and RT separate methods showed generally similar trends. With the exception of a few abnormal ECGs, the bulk method can be regarded as the standard one because it provides a clear visual representation of TCG results.

### Relationship between TCG and 12-lead ECG

TCG can be applied to any lead of the ECG. Basically, the leads along the main electrical axis of the heart, such as II and CM5, are easy to understand because the directions of depolarization and repolarization are straightforward. Also, the thoracic leads from V3 to V6 have a similar relationship due to the proximity of the electrodes to the heart.

The TCG results for ECGs recorded from specific electrodes can be analyzed in accordance with the corresponding association between electrodes and regions of the heart in conventional ECG interpretation methods. ECG leads, such as AVL V1 V2, deviate from the major axis of cardiac electrical conduction and the spatial relationship between dipoles during depolarization and repolarization is complex; it cannot be simulated with four CDFs and must be represented with additional CDFs, which are described in the Supporting Information (Extensions to complex ECGs, S2 Method).

### Limitations of TCG metrics and myocardial action potentials

The model of TCG uses a point process as in conventional cardiac electrophysiology (i.e., it is a dipole model); it is not clear at this time whether the results obtained by TCG are consistent with actual collective myocardial AP transitions. It should be noted that the plateau phase (phase 2) of the AP is not horizontal and repolarization proceeds slowly in phase 2 with variations between myocytes, which affects the timing of the repolarization transition (S1 Fig). As a result, the repolarization timing of the CDF does not always coincide with the timing of the acute descending phase (phase 3) of the AP waveform. In particular, in the case of a prominent decline in the plateau phase (phase 2) of the AP, such as in ischemia, μTn (the CDF of cathodal repolarization) is likely to be positioned anterior to the normal location[12,39]. Further studies involving animal experiments and cardiac simulations are needed in order to confirm the consistency between the TCG metrics and the collective AP transitions.

### Application of TCG for predicting myocardial ischemia

TCG explicitly expresses the relationship between the ECG and collective myocardial AP transition state in terms of statistical measures. TCG may have applications to clinical ECG because it has the potential to capture pathological distortions that are not adequately captured by conventional methods. TCG calculates a time series of three parameters for each of the four CDFs for each beat and each lead, and pathological strain is explicitly represented in the tensor. This tensor differs from a physics tensor (such as reciprocity, strain or stress); rather, it is a representation of multi-order data. The information about the temporal and spatial relationships due to the regulated activity of the myocardial collective can be expanded into a definite form as a tensor from the ECG. Using TCG, the probability density distribution (mean μ, variance σ and weightk) of the CDFs can be obtained for the transition process of collective myocardial APs for each beat, making it possible to statistically test for variations and abnormalities in the AP transitions for each beat. For example, the TCG results for every heartbeat of an individual participant can be statistically tested against the standard values of a normal control group.

We have shown that TCG can make an early detection of myocardial ischemia. Myocardial ischemia and risk of fatal arrhythmia are respectively identified by ST changes and prolonged QT intervals in ECG measurements. However, tiny ST changes in an ECG might often be overlooked as indicating the presence of myocardial ischemia. In addition, QT interval prolongation is detected by performing manual measurements after the ECG recording, meaning that it is difficult to confirm the presence of the QT prolongation in a real-time ECG recording. In the present study, as shown in Fig 4C, marked ST elevation was visually manifested by a ST segment elevation +2.0mm after the first pre-dilation for stenotic lesion of the coronary artery by balloon inflation prior to stent implantation. However, such ST changes are confirmed approximately 10 sec after balloon inflation. On the other hand, our TCG analysis revealed that significant μRTn and μRTp changes at the time of balloon inflation prior to ST segment elevation. Moreover, it was able to reveal repolarization abnormalities. It is known that repeated balloon inflation diminishes ST segment elevation because of ischemic preconditioning [47]. Indeed, the 2^nd^ balloon inflation for stent implantation revealed a mild ST segment elevation compared with the first balloon inflation. On the other hand, the μRTn deviation showed the same response as the first balloon inflation indicating that μRTn is more sensitive than μRTp to myocardial ischemia. The present study thus indicates that TCG analysis offers real-time, accurate and sensitive quantitative assessments for the evaluation of ST segment deviations and QT intervals associated with myocardial ischemia.

### Clinical implications of TCG for predicting life-threatening arrhythmia

Non-sustained ventricular tachycardia (VT) and R on T type of premature ventricular contraction (PVC) are well-known warning arrhythmia that might lead to VT or VF [48] Here, it would be ideal to be able to predict life-threatening arrhythmia by conducting an ECG analysis prior to initiation of VT or VF during sinus rhythm. Microvolt T-wave alternans, an oscillation in T-wave morphology, is associated with increased susceptibility to VT and VF [26]. Therefore, repolarization abnormalities are an important sign of the development of VT and VF. Indeed, in the patients with Brugada syndrome or early repolarization syndrome, the heterogeneity between the epicardial and endocardial sides during the myocardial repolarization is considered to be one of the mechanisms leading to the onset of lethal ventricular arrhythmias [49]. However, tiny changes in the repolarization phase between the epicardium and endocardium did not manifest as ECG changes. TCG analysis made it possible to detect ventricular repolarization abnormalities prior to VF onset, which were difficult to identify visually from ECG. In addition, TCG could be used to evaluate beat-to-beat dynamic repolarization changes between the epi- and endocardium of the ventricle that do not appear in 12-lead ECG. TCG analysis may thus enable earlier detection of abnormalities before manifestation of ECG changes in the various cardiovascular diseases. In addition, TCG is calculated from real-time beat-to-beat ECG changes. It might thus be applicable to ECG monitoring for early detection of myocardial ischemia and ECG changes warning of life-threatening arrhythmias. Further study will be needed to validate and elucidate the TCG analysis for clinical applications.

### Single heartbeat abnormal detection by TCG

It is also possible to test the beat-to-beat statistical difference and/or calculate the mathematical distance between the stable normal condition and unstable abnormal states of heart disease. The MD can be used as a method for determining the statistical distances. For example, the MD from the observed ECG can be determined by using a certain number of stable-phase TCG results (a reference distribution) as an index of evaluation.

### Eliminating noise and distortion from ECGs

ECGs are prone to waveform distortion induced by factors like body motion and electromagnetic interference, particularly from commercial AC waves. Addressing this issue requires effective noise elimination or suppression techniques. Conventionally, spatial filtering, which restricts frequency bandwidth, has been employed for noise and distortion reduction in ECGs. However, this method has drawbacks, including waveform distortion, attenuation, and phase shift.

In engineering, solving the wave equation by utilizing sinusoidal waves is a common approach to processing electromagnetic signals to eliminate noise and facilitate efficient analysis of frequency, amplitude, and phase. In our case, a similar beneficial noise reduction and signal enhancement effect may be had by applying the difference equation derived from the cumulative density function of TCG to electrocardiogram analysis.

### Noninvasive assessment of action potential duration (APD)

The APD is measured from unipolar electrograms through electrodes from catheters directly attached to the heart. This invasive procedure presents many challenges. A promising noninvasive alternative involves using μRTn and μRTp derived from TCG to represent the duration of collective action potentials. This noninvasive approach would offer potential clinical utility for estimating APD.

Additionally, when TCG is used, intracardiac unipolar ECGs may show differences from body surface ECGs in the anode p:end, cathode n:epi relationship that are contingent on instrument settings. For instance, μRTp obtained through TCG may signify the duration of global action potentials, while μRTn may reflect local action potential duration. Comprehensive studies are thus warranted to elucidate these relationships and refine noninvasive APD estimation methods.

### Education and research tools for ECG

ECG is used not only by medical professionals, but also by non-medical researchers and the general public. Because of the widespread use of ECG and the necessity for understanding its mechanisms, there is a demand for effective educational and research tools. We believe that TCG is a promising solution for educational and research contexts for its ability to vividly illustrate the intricate relationship between myocardial action potentials and ECG.

### Limitation

In this study, we examined the accuracy of fitting ECG data using TCG analysis. We applied TCG analysis to ECG data from 699 participants on PhysioNet, considered healthy subjects, and to only a few cases of specific heart diseases, including myocardial ischemia and ventricular arrhythmia. Therefore, further study with a larger number of case samples is required to understand the clinical significance of the various measurements obtained by TCG.

## Supporting information

S1 Method Equations of model of collective ventricular muscle AP transitions as a polar sign-marked point process. (DOCX)

S2 Method Extensions to complex ECGs. (DOCX)

S3 Method Mahalanobis distance. (DOCX)

S1 Fig (Graphical Abstract). Equation and metric of the ECG with Cumulative Density Function (CDF) combination. ECG is modeled by the equation involving the difference between two cumulative distribution functions (CDFs) and is used to fit the R and T waves separately by using the least squares method. The 4 CDFs (Optional +2 CDFs were added for delta wave or deep S wave if necessary, inset box) represent the frequency distribution of occurrences of depolarization and repolarization of a population of myocardial cells and are used to measure the synchrony and time shift of them (Upper Figures). The standard deviation(σ), mean(μ), weights(κ), and level(β) of each CDF are statistical measures of the anode-cathode of the cardiac EMF dipole in the depolarization and repolarization phases, respectively, which, when combined with the time series of beats (n) and the number of ECG lead channels (12, in case of the standard 12 leads), form a fourth-order tensor (Lower Figures). TCG: Tensor cardiography.
(TIF)

S2 Fig Scatter plots of S1A and S1B Table. (TIF)

S3 Fig Animation showing TCG of continuous sinus rhythm (10 beats of Fig 5A–5D, Case 2, Early repolarization syndrome). (MP4)

S4 Fig TCG (bulk method) results for I, II, and V2 of standard 12-lead ECG. (TIF)

S1 Table **A,** The correlation coefficients between the TCG parameters with relation to time (interval), QT, and R-R interval (RRI). **B**, The correlation coefficients for the TCG parameters related to potentials. (The results of TCG performed on 699 participants selected from the ECG data of the PhysioNet Autonomic Aging database).
(TIF)

S2 Table TCG parameters in the S4 Fig (The results of bulk method, I, II, and V2 of standard 12-lead ECG). (TIF)

S3 Table Parameters of TCG analysis (S1 Fig), CDF: Cumulative Density Function. *Delta: Optional CDFs for complexed waves such as Delta waves (WPW syndrome), J waves (Brugada syndrome), Deep Q or S waves, rsR’ pattern (right Bundle Branch Block).
(TIF)
