# Supplementary material for: Tensor cardiography: A novel ECG analysis of deviations in collective myocardial action potential transitions based on point processes and cumulative distribution functions
Source: PLOS Digit Health. 2024 Aug 8;3(8):e0000273. doi: 10.1371/journal.pdig.0000273 (PMC11309480; doi:10.1371/journal.pdig.0000273)
Supplement: S1 Method — (DOCX) [file pdig.0000273.s001.docx]

**S1 Method.**

**Equations of model of collective ventricular muscle AP transitions as a polar sign-marked point process**

Expressions (1) and (2) below are respectively the Gaussian distribution representing the probability density of depolarization time series of the anodic, positive potential (inner layer) of the ventricular muscle, with mean μ_p_ and standard deviation σ_p_ (variance is σ_p_²) and the Gaussian distribution of the unimodal distribution representing the probability density of depolarization time series of the cathodic, negative potential of the ventricular muscle, with mean μ_n_ and standard deviation σ_n_ (variance is σ_n_²), and x representing time.

$$\frac{1}{\sqrt{2\pi{\sigma_{p}}^{2}}}exp\left( -\frac{\left( x-\mu_{p} \right)^{2}}{2{\sigma_{p}}^{2}} \right) \cdots(1)$$

$$\frac{1}{\sqrt{2\pi{\sigma_{n}}^{2}}}exp\left( -\frac{\left( x-\mu_{n} \right)^{2}}{2{\sigma_{n}}^{2}} \right) \cdots(2)$$

Equation (3) is the cumulative distribution function (CDF) of Expression (1) and Equation (4) is the CDF) of Expression (2), where "erf" is the sigmoid function.

$$f_{p}\left( x \right)=\frac{1}{2}\left( 1+erf\left( \frac{x-\mu_{p}}{\sqrt{2{\sigma_{p}}^{2}}} \right) \right) \cdots(3)$$

$$f_{n}\left( x \right)=\frac{1}{2}\left( 1+erf\left( \frac{x-\mu_{n}}{\sqrt{2{\sigma_{n}}^{2}}} \right) \right) \cdots(4)$$

The function of subtracting the second CDF (4) from the first CDF (3) is

$$f\left( x \right)=f_{p}\left( x \right)-f_{n}\left( x \right)=\frac{1}{2}\left( 1+erf\left( \frac{x-\mu_{p}}{\sqrt{2{\sigma_{p}}^{2}}} \right) \right)- \frac{1}{2}\left( 1+erf\left( \frac{x-\mu_{n}}{\sqrt{2{\sigma_{n}}^{2}}} \right) \right) \cdots(5)$$

When the CDF $f_{p}\left( x \right)$ in the inner layer and the CDF $f_{n}\left( x \right)$ in the outer layer are weighted and the difference is taken, a weight *k_p_* is given to $f_{p}\left( x \right)$ and a weight *k_n_* is given to $f_{n}\left( x \right)$.

$$f\left( x \right)={{k_{p}f}_{p}\left( x \right)-k_{n}f_{n}\left( x \right)=k}_{p}\frac{1}{2}\left( 1+erf\left( \frac{x-\mu_{p}}{\sqrt{2{\sigma_{p}}^{2}}} \right) \right)-k_{n} \frac{1}{2}\left( 1+erf\left( \frac{x-\mu_{n}}{\sqrt{2{\sigma_{n}}^{2}}} \right) \right)\cdots(6)$$

The target cardiac potential time waveform F(x) is approximated with the approximate time waveform f(x), which is the difference between the first and second CDF. The least squares method (L2-norm) (7) can be used to make this approximation.

Least squares fit

$F\left( x \right)$ Observed ECG data (target ECG data)

$f\left( x \right)$ Time sequence probability density function

Minimize the sum of squares of the residuals (L2-norm)

$$\mathrm{Minimize} \sum_{x=1}^{n} \left( F\left( x \right)-f\left( x \right) \right)^{2} \cdots(7)$$

The depolarization section waveform (R wave) $f_{R}\left( x \right)$ is approximated by the difference between the CDF of the positive section and that of the negative section. Equation (8) expresses the positive CDF $f_{Rp}\left( x \right)$and Equation (9) the negative CDF $f_{Rn}\left( x \right)$. The depolarization interval waveform $f_{R}\left( x \right)$ of the ECG is approximated by the time waveform of Equation (10), which subtracts the negative CDF $f_{Rn}\left( x \right)$ from the positive CDF $f_{Rp}\left( x \right)$. In these equations, the parameters that represent the characteristics of the depolarization interval waveform (R wave) are the mean μ_Rp_ and standard deviation σ_Rp_, which identify the positive CDF, and the mean μ_Rn_ and standard deviation σ_Rn_, which identify the negative CDF (11).

$F_{R}\left( x \right)$ Observed R wave data (target R wave data)

$f_{R}\left( x \right)$ Time sequence probability density function for R wave

$$f_{Rp}\left( x \right)=\frac{1}{2}\left( 1+erf\left( \frac{x-\mu_{Rp}}{\sqrt{2{\sigma_{Rp}}^{2}}} \right) \right) \cdots(8)$$

$$f_{Rn}\left( x \right)=\frac{1}{2}\left( 1+erf\left( \frac{x-\mu_{Rn}}{\sqrt{2{\sigma_{Rn}}^{2}}} \right) \right) \cdots(9)$$

$$f_{R}\left( x \right)=f_{Rp}\left( x \right)-f_{Rn}\left( x \right)=\frac{1}{2}\left( 1+erf\left( \frac{x-\mu_{Rp}}{\sqrt{2{\sigma_{Rp}}^{2}}} \right) \right)- \frac{1}{2}\left( 1+erf\left( \frac{x-\mu_{Rn}}{\sqrt{2{\sigma_{Rn}}^{2}}} \right) \right)\cdots(10)$$

The depolarization section waveform (i.e., R wave) $f_{R}\left( x \right)$ is found by taking the difference between the weighted positive CDF $f_{Rp}\left( x \right)$ and the negative CDF $f_{n}\left( x \right)$:

$$f_{R}\left( x \right)={{k_{Rp}f}_{Rp}\left( x \right)-k_{Rn}f_{n}\left( x \right)=k}_{Rp}\frac{1}{2}\left( 1+erf\left( \frac{x-\mu_{Rp}}{\sqrt{2{\sigma_{Rp}}^{2}}} \right) \right)-k_{Rn} \frac{1}{2}\left( 1+erf\left( \frac{x-\mu_{Rn}}{\sqrt{2{\sigma_{Rn}}^{2}}} \right) \right), (11)$$

where is k_Rp_ is the weight of $f_{Rp}\left( x \right)$ and is k_Rn_ the weight of $f_{Rn}\left( x \right)$, and one minimizes

$$\sum_{x=R1}^{Rn} \left( F_{R}\left( x \right)-f_{R}\left( x \right) \right)^{2} \cdots(12)$$

The R wave is a ventricular myocardium sequential depolarization (OFF to ON, S1 Fig), while the T wave is the corresponding repolarization (ON to OFF). Thus, the T wave corresponds the opposite switching to the R wave on the time axis.

Therefore, for the repolarization interval waveform (T wave), two inverse CDFs , wherein the CDF is subtracted from 1 are used, and the difference or weighted difference between the positive inverse CDF ${f'}_{Tp}\left( x \right)$ and the negative inverse CDF ${f'}_{Tn}\left( x \right)$ is computed.

$${f^{'}}_{Tp}\left( x \right)=1-f_{Tp}\left( x \right)=1-\frac{1}{2}\left( 1+erf\left( \frac{x-\mu_{Tp}}{\sqrt{2{\sigma_{Tp}}^{2}}} \right) \right) \cdots(13)$$

$${f^{'}}_{Tn}\left( x \right)=1-f_{Tn}\left( x \right)=1-\frac{1}{2}\left( 1+erf\left( \frac{x-\mu_{Tn}}{\sqrt{2{\sigma_{Tn}}^{2}}} \right) \right) \cdots(14)$$

$F_{T}\left( x \right)$ Observed T-wave data (target T-wave data)

$f_{T}\left( x \right)$ Time sequence probability density function for T wave

${f'}_{T}\left( x \right)$ Reversed time sequence probability density function for T wave

$$f_{T}\left( x \right)={f^{'}}_{Tp}\left( x \right)-{f^{'}}_{Tn}\left( x \right)=\left( 1-f_{Tp}\left( x \right) \right)-\left( 1-f_{Tn}\left( x \right) \right)=f_{Tn}\left( x \right)-f_{Tp}\left( x \right)=\frac{1}{2}\left( 1+erf\left( \frac{x-\mu_{Tn}}{\sqrt{2{\sigma_{Tn}}^{2}}} \right) \right)-\frac{1}{2}\left( 1+erf\left( \frac{x-\mu_{Tp}}{\sqrt{2{\sigma_{Tp}}^{2}}} \right) \right) \cdots(15)$$

$$f_{T}\left( x \right)={{k_{Tp}f^{'}}_{Tp}\left( x \right)-k_{Tn}{f^{'}}_{Tn}\left( x \right) =k_{Tp}\left( 1-f_{Tp}\left( x \right) \right)-k_{Tn}\left( 1-f_{Tn}\left( x \right) \right)={k_{Tn}f_{Tn}\left( x \right)-k}_{Tp}f_{Tp}\left( x \right)+k_{Tp}{-k}_{Tn}=k_{Tn} \frac{1}{2}\left( 1+erf\left( \frac{x-\mu_{Tn}}{\sqrt{2{\sigma_{Tn}}^{2}}} \right) \right)-k_{Tp} \frac{1}{2}\left( 1+erf\left( \frac{x-\mu_{Tp}}{\sqrt{2{\sigma_{Tp}}^{2}}} \right) \right)+k_{Tp}-k_{Tn}} \cdots(16)$$

Minimize

$$\sum_{x=T1}^{Tn} \left( F_{T}\left( x \right)-f_{T}\left( x \right) \right)^{2} \cdots(17)$$

The repolarization section waveform (T wave) is approximated by the function ${f'}_{Tp}\left( x \right)$(inverse positive CDF), which is the difference between the positive CDF $f_{Tp}\left( x \right)$ from 1 (Equation (13)), and the function ${f'}_{Tn}\left( x \right)$ (inverse negative CDF), which is the difference of the negative CDF $f_{Tn}\left( x \right)$ from 1 (Equation (14)).The repolarization time waveform is approximated by Equation (15), which subtracts the inverse negative CDF ${f'}_{Tn}\left( x \right)$ from the inverse positive CDF ${f'}_{Tp}\left( x \right)$.

When the repolarization interval waveform (T wave) is approximated by the weighted difference between the inverse positive CDF ${f'}_{Tp}\left( x \right)$ and inverse negative CDF ${f'}_{Tn}\left( x \right)$, the repolarization time waveform is approximated by the approximate time waveform of Equation (16), in which the inverse negative CDF ${f'}_{Tn}\left( x \right)$ multiplied by the weight k_Tn_ is subtracted from the inverse positive CDF${f'}_{Tp}\left( x \right)$ multiplied by the weight k_Tp_.

The difference between the observed ECG R wave and the CDF, $f_{Rp}\left( x \right)$-$f_{Rn}\left( x \right)$, and the difference between the T wave and $f_{Tn}\left( x \right)$-$f_{Tp}\left( x \right)$ are minimized, and the parameters of the four CDFs are obtained.

The weights k of the CDFs of depolarization (R wave) and repolarization (T wave) are determined using two methods: 1) a method of determining the four independent CDFs’ value of k (RT separation method, Fig 2A) and (2) a method of determining k under the condition that weights k of the depolarization (R wave) and repolarization (T wave) CDFs are equal, which means the positive pair, $f_{Rp}\left( x \right)$and ${f'}_{Tp}\left( x \right)$,and negative pair, $f_{Rn}\left( x \right)$ and ${f'}_{Tn}\left( x \right)$, are connected in the plateau (RT bulk method, Fig 2B).

(RT bulk method, the points between the R peak and the R peak + 60 msec that are lower than the ST junction point are excluded from the fitting. This is to prevent the s downward peak from adversely affecting the fitting.)

The computer program for solving Eqs. (12) and Eqs. (17) calculated all four CDFs parameters and the CDFs intervals as well as conventional ECG variables including RR interval, QT time, and ST level by using the least squares method.

μRTp, the interval between μRp and μTp, represents the average duration of positive collective APs, and μRTn, the interval between μRn and μTn, represents the duration of negative collective APs (Fig 2B).

The results of this analysis can be expressed as a fourth-order tensor, with three dimensions (ECG leads, CDFs, and a parameter) and one dimension in time. For example, the number of ECG leads (e.g., I, II, and V2), 4CDFs, the parameters of the distribution (average μ, standard deviation σ, weight k, baseline level β) and time of heartbeat form the tensor <3,4,4,1> data (S3 Fig).
