# Supplementary material for: Tensor cardiography: A novel ECG analysis of deviations in collective myocardial action potential transitions based on point processes and cumulative distribution functions
Source: PLOS Digit Health. 2024 Aug 8;3(8):e0000273. doi: 10.1371/journal.pdig.0000273 (PMC11309480; doi:10.1371/journal.pdig.0000273)
Supplement: S2 Method — (DOCX) [file pdig.0000273.s002.docx]

**S2 Method.**

**Extensions to complex ECGs**

ECGs with abnormal waves (e.g., delta and J waves), T-wave diversity (e.g., with inflection points) and complicated excitation propagation projected leads (e.g., aVL, V1, and V2) cannot be represented completely with four CDFs. In such cases, more CDFs are added to increase expressiveness.

For example, abnormal waves that appear around R and T waves, such as delta and J waves, or with irregularities or inflection points (subtle wave) not normally seen in ST and T waves, can be handled by adapting the same process previously described for R and T waves to abnormal waves. For example, when approximating Δ waves $F_{D}$ which appear between R and T waves, the $f_{D}$ difference between the CDF $f_{Dp}\left( x \right)$ and CDF $f_{Dn}\left( x \right)$ can be approximated by the time waveform of Equation (18), and the abnormal waveforms obtained as parameters that represent the characteristics of the waves.

$$f_{D}\left( x \right)={{-k}_{Dn}f_{Dn}\left( x \right)+k}_{Dp}f_{Dp}\left( x \right)$$

=$k_{Dp}\frac{1}{2}\left( 1+erf\left( \frac{x-\mu_{Dp}}{\sqrt{2{\sigma_{Dp}}^{2}}} \right) \right)-k_{Dn} \frac{1}{2}\left( 1+erf\left( \frac{x-\mu_{Dn}}{\sqrt{2{\sigma_{Dn}}^{2}}} \right) \right)$⋯(18)

Minimize

$$\sum_{x=D1}^{Dn} \left( F_{D}\left( x \right)-f_{D}\left( x \right) \right)^{2} \cdots(19)$$
