# Supplementary material for: Tensor cardiography: A novel ECG analysis of deviations in collective myocardial action potential transitions based on point processes and cumulative distribution functions
Source: PLOS Digit Health. 2024 Aug 8;3(8):e0000273. doi: 10.1371/journal.pdig.0000273 (PMC11309480; doi:10.1371/journal.pdig.0000273)
Supplement: S3 Method — (DOCX) [file pdig.0000273.s003.docx]

**S3 Method.**

**Mahalanobis distance**

For s multivariate analysis of variance (MANOVA) or a Hotelling T^2^ test, the Mahalanobis distance (MD) is the multivariate counterpart of d:

$$d=\sqrt{\left( x-\mu\right)^{T}\Sigma^{-1}\left( x-\mu\right)}$$

where$x$ and $\mu$ are the mean vectors of the two groups in the comparison, $\left( x-\mu\right)$ is the transposed mean vector differences $\Sigma^{-1}$is the inverse of the pooled covariance matrix [1].

**References**

1 Fan X, Konold TR. Statistical Significance Versus Effect Size. In: Peterson P, Baker E, McGaw B, editors. International Encyclopedia of Education (Third Edition). Oxford: Elsevier; 2010. pp. 444–450. doi:10.1016/B978-0-08-044894-7.01368-3
