# Supplementary figures and images for: Tensor cardiography: A novel ECG analysis of deviations in collective myocardial action potential transitions based on point processes and cumulative distribution functions
Source: PLOS Digit Health. 2024 Aug 8;3(8):e0000273. doi: 10.1371/journal.pdig.0000273 (PMC11309480; doi:10.1371/journal.pdig.0000273)

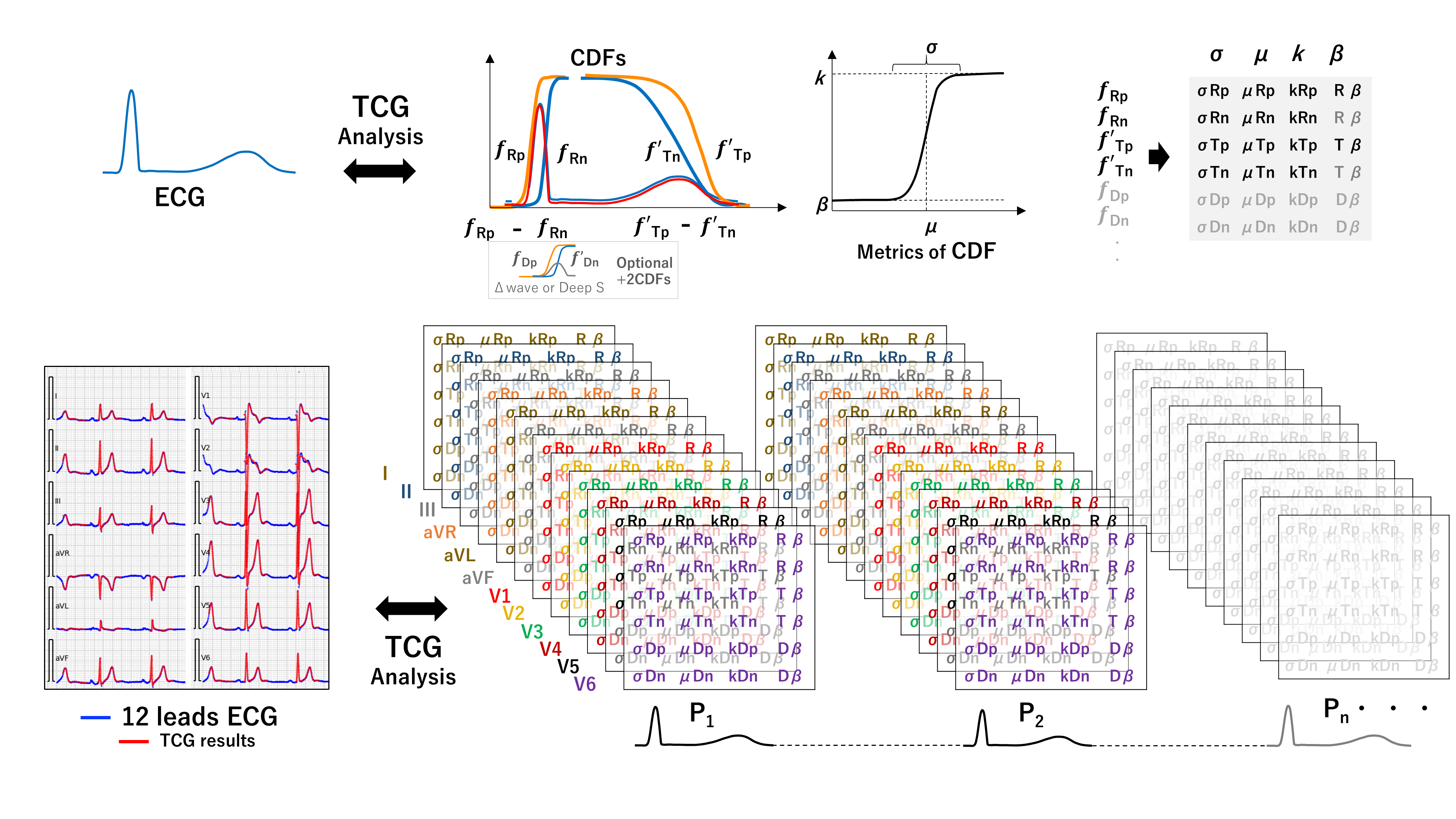

Supplement: S1 Fig — ECG is modeled by the equation involving the difference between two cumulative distribution functions (CDFs) and is used to fit the R and T waves separately by using the least squares method. The 4 CDFs (Optional +2 CDFs were added for delta wave or deep S wave if necessary, inset box) represent the frequency distribution of occurrences of depolarization and repolarization of a population of myocardial cells and are used to measure the synchrony and time shift of them (Upper Figures). The standard deviation(σ), mean(μ), weights(κ), and level(β) of each CDF are statistical measures of the anode-cathode of the cardiac EMF dipole in the depolarization and repolarization phases, respectively, which, when combined with the time series of beats (n) and the number of ECG lead channels (12, in case of the standard 12 leads), form a fourth-order tensor (Lower Figures). TCG: Tensor cardiography. (TIF) [file pdig.0000273.s004.TIF]

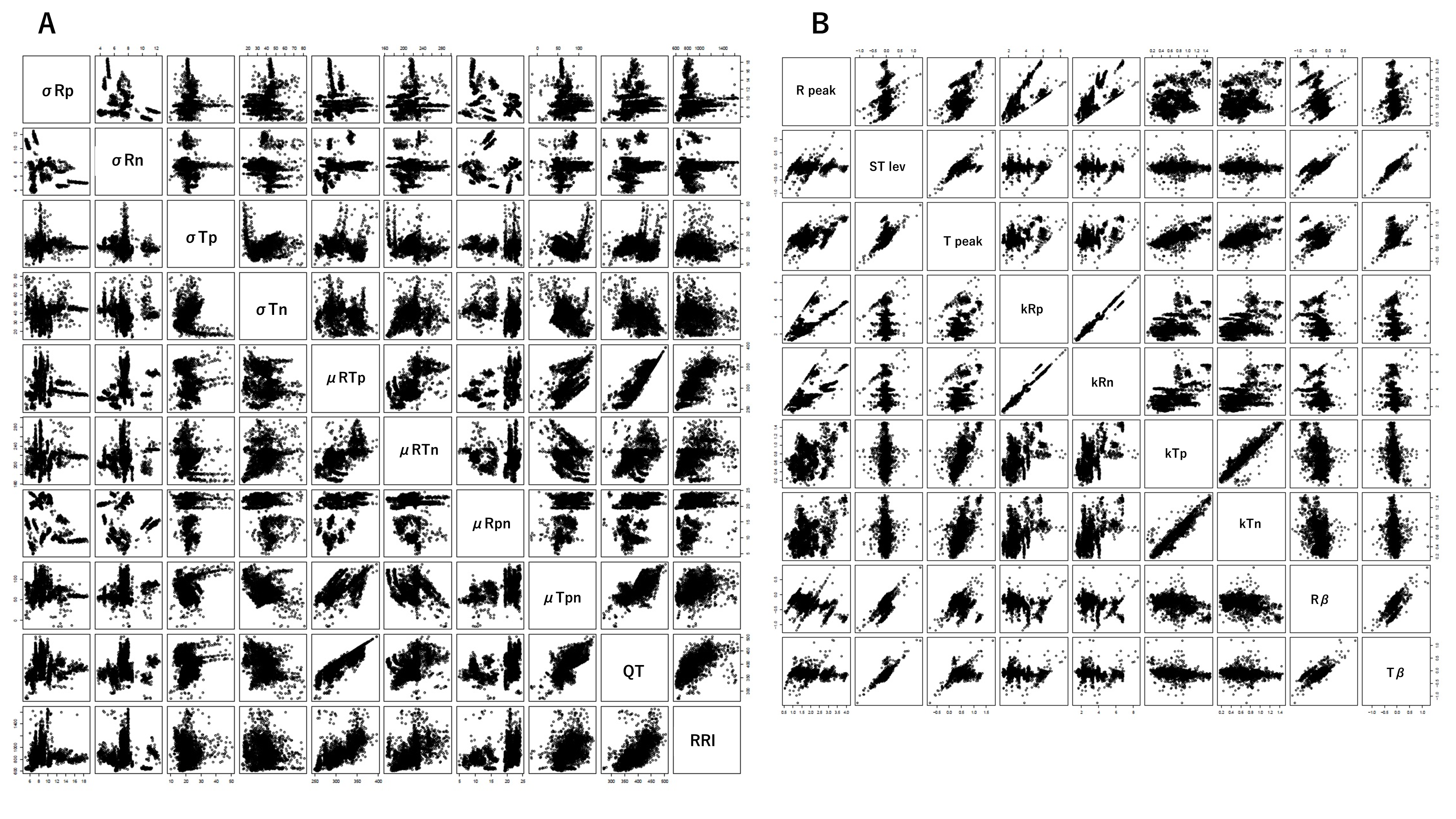

Supplement: S2 Fig — (TIF) [file pdig.0000273.s005.TIF]

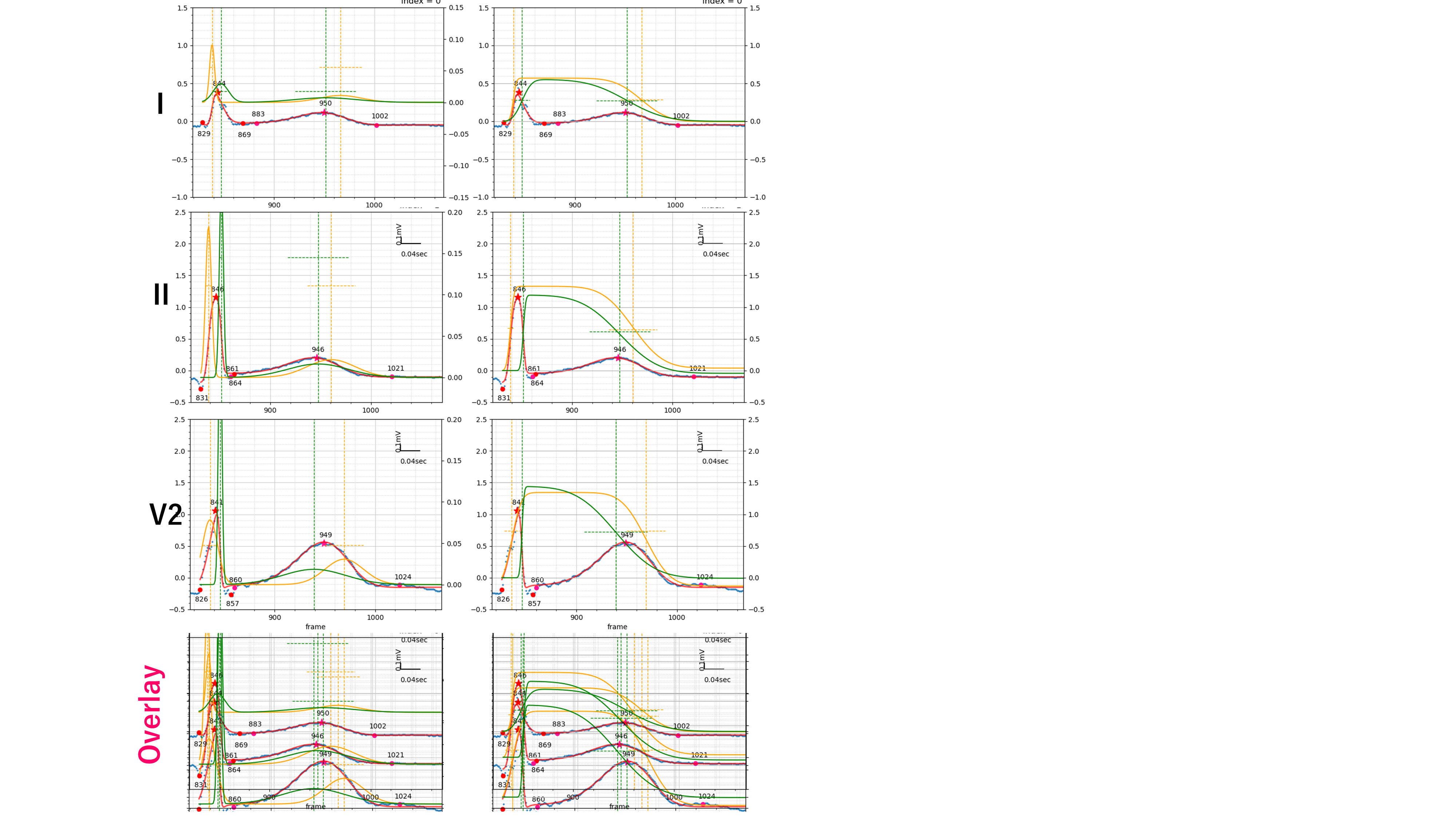

Supplement: S4 Fig — (TIF) [file pdig.0000273.s007.TIF]

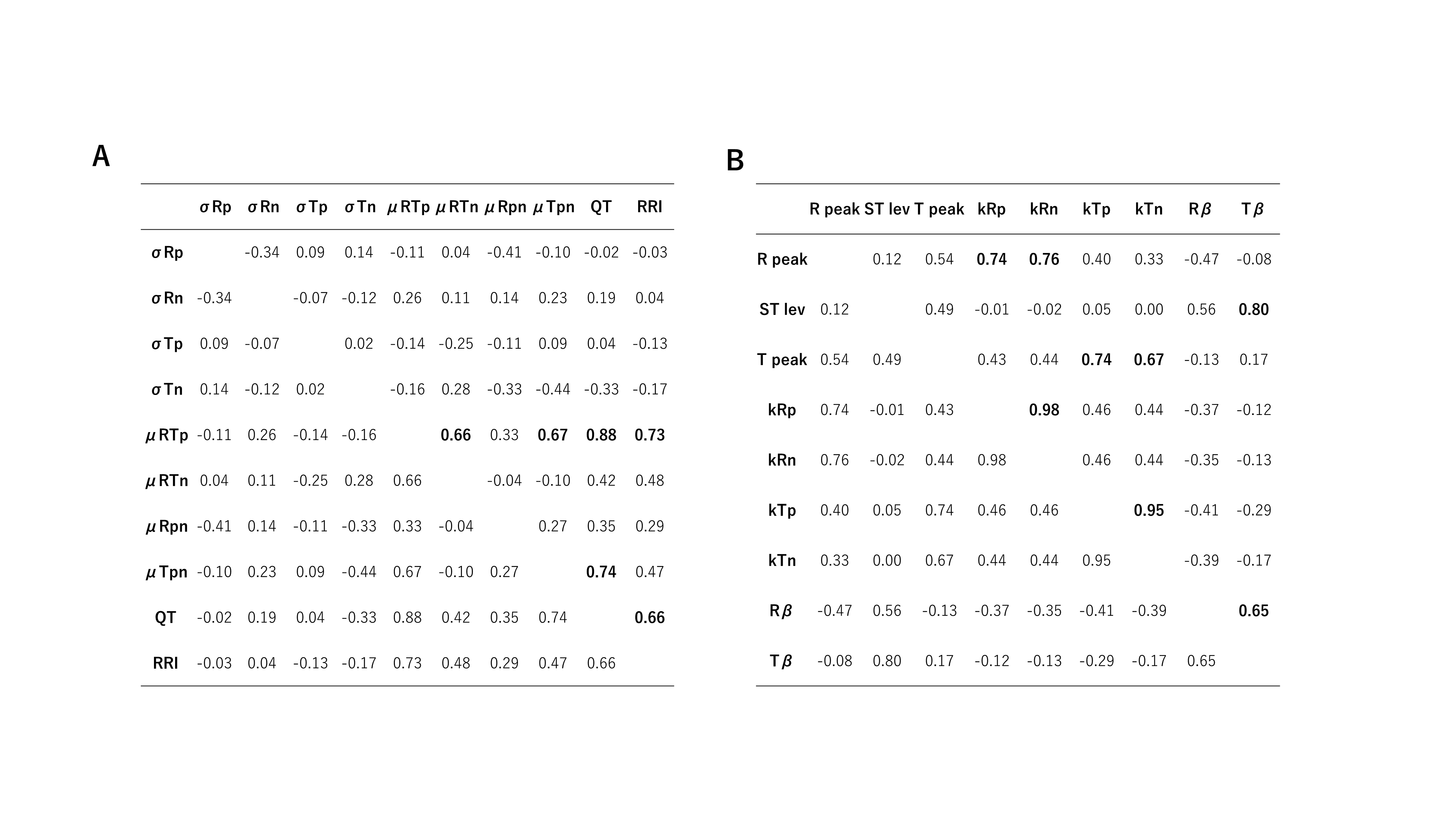

Supplement: S1 Table — A, The correlation coefficients between the TCG parameters with relation to time (interval), QT, and R-R interval (RRI). B, The correlation coefficients for the TCG parameters related to potentials. (The results of TCG performed on 699 participants selected from the ECG data of the PhysioNet Autonomic Aging database). (TIF) [file pdig.0000273.s008.TIF]

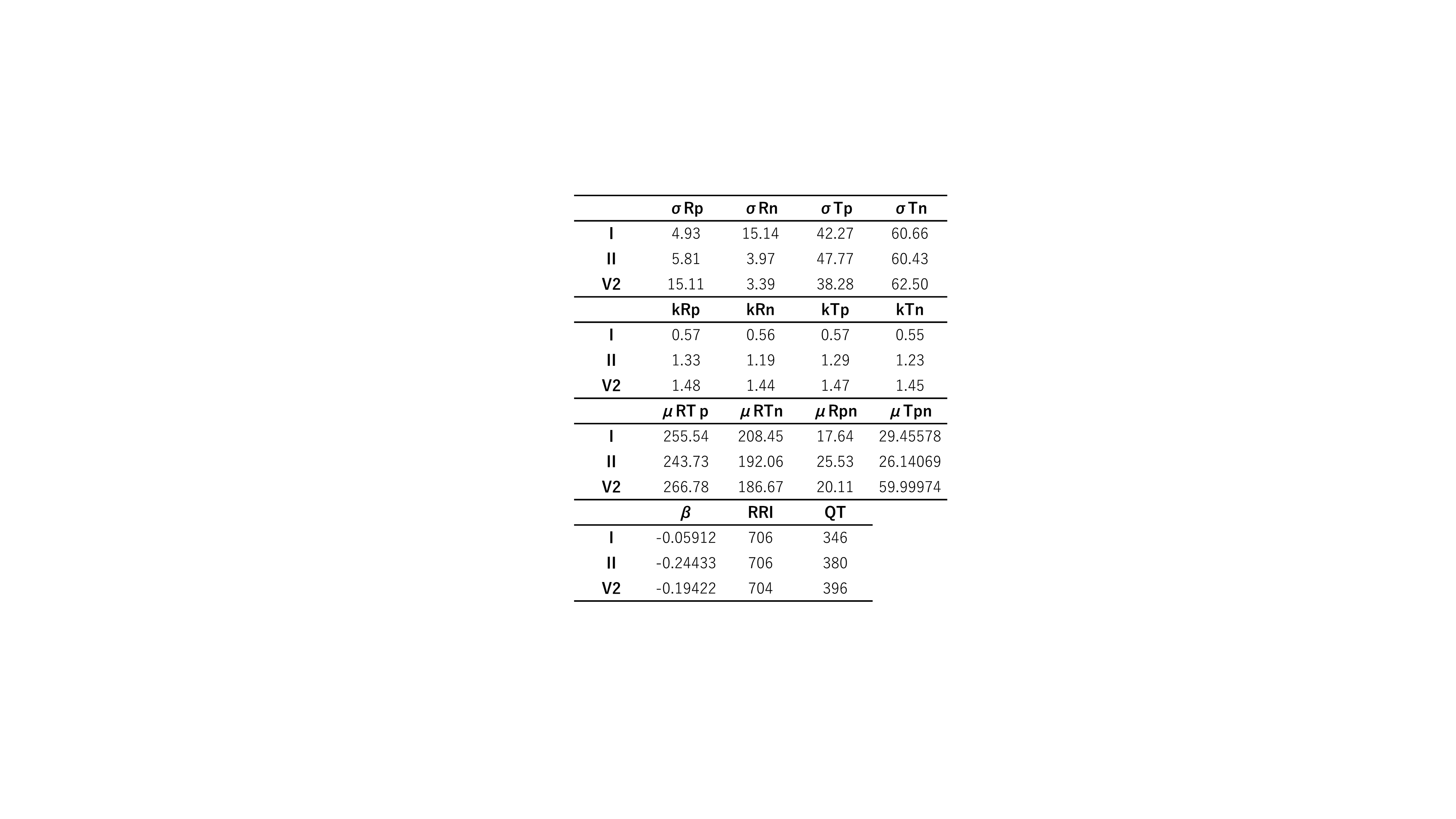

Supplement: S2 Table — (TIF) [file pdig.0000273.s009.TIF]

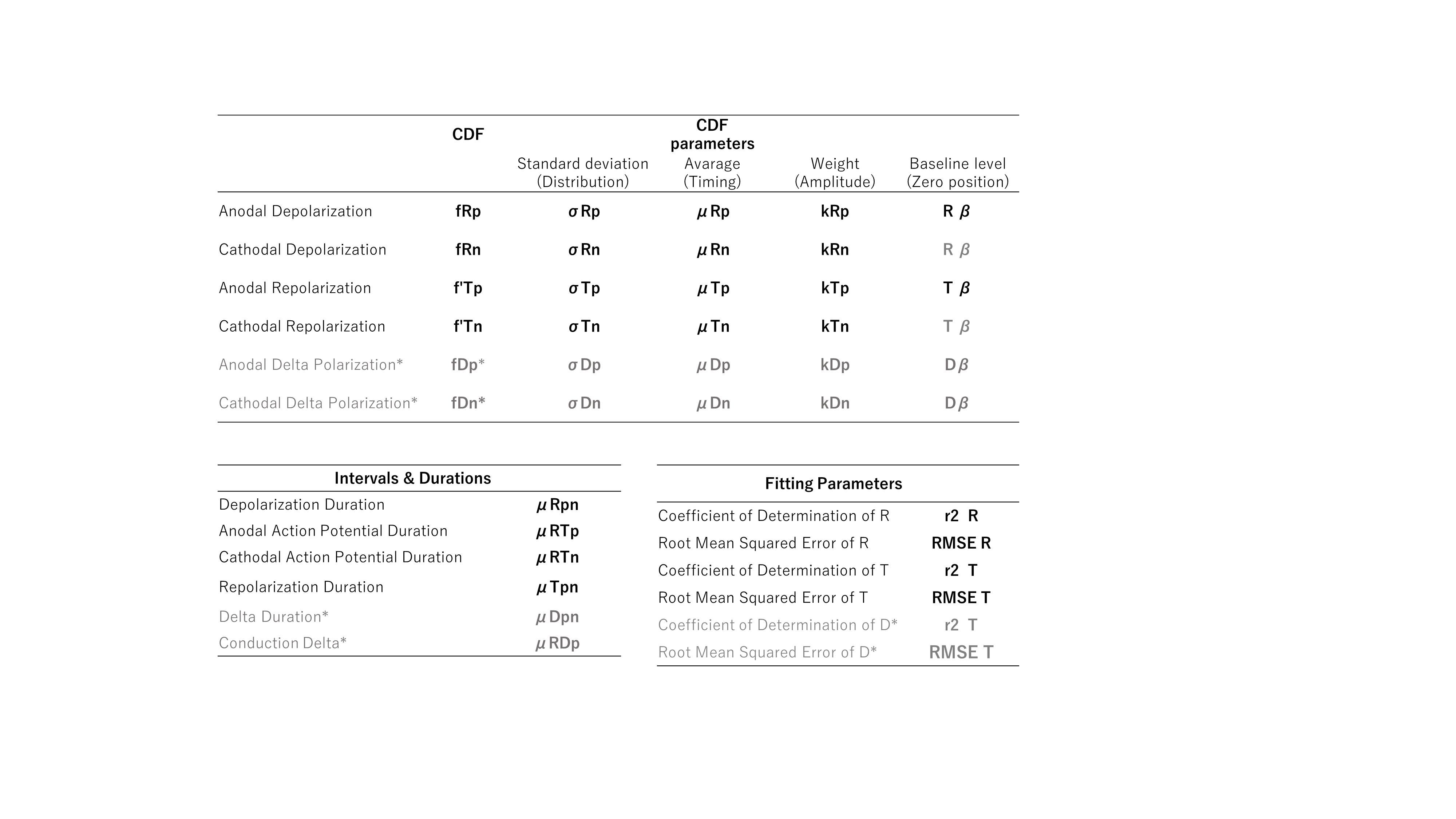

Supplement: S3 Table — *Delta: Optional CDFs for complexed waves such as Delta waves (WPW syndrome), J waves (Brugada syndrome), Deep Q or S waves, rsR’ pattern (right Bundle Branch Block). (TIF) [file pdig.0000273.s010.TIF]
